# Non-equivalent, but still valid: Establishing the construct validity of a consumer fitness tracker in persons with multiple sclerosis

**DOI:** 10.1371/journal.pdig.0000171

**Published:** 2023-01-25

**Authors:** Ashley Polhemus, Chloé Sieber, Christina Haag, Ramona Sylvester, Jan Kool, Roman Gonzenbach, Viktor von Wyl

**Affiliations:** 1 Epidemiology, Biostatistics, and Prevention Institute, University of Zurich, Zurich, Switzerland; 2 Institute for Implementation Science in Health, University of Zurich, Zurich, Switzerland; 3 Research Department Physiotherapy, Rehabilitation Centre, Valens, Switzerland; McGill University, CANADA

## Abstract

Tools for monitoring daily physical activity (PA) are desired by persons with multiple sclerosis (MS). However, current research-grade options are not suitable for longitudinal, independent use due to their cost and user experience. Our objective was to assess the validity of step counts and PA intensity metrics derived from the Fitbit Inspire HR, a consumer-grade PA tracker, in 45 persons with MS (Median age: 46, IQR: 40–51) undergoing inpatient rehabilitation. The population had moderate mobility impairment (Median EDSS 4.0, Range 2.0–6.5). We assessed the validity of Fitbit-derived PA metrics (Step count, total time in PA, time in moderate to vigorous PA (MVPA)) during scripted tasks and free-living activity at three levels of data aggregation (minute, daily, and average PA). Criterion validity was assessed though agreement with manual counts and multiple methods for deriving PA metrics via the Actigraph GT3X. Convergent and known-groups validity were assessed via relationships with reference standards and related clinical measures. Fitbit-derived step count and time in PA, but not time in MVPA, exhibited excellent agreement with reference measures during scripted tasks. During free-living activity, step count and time in PA correlated moderately to strongly with reference measures, but agreement varied across metrics, data aggregation levels, and disease severity strata. Time in MVPA weakly agreed with reference measures. However, Fitbit-derived metrics were often as different from reference measures as reference measures were from each other. Fitbit-derived metrics consistently exhibited similar or stronger evidence of construct validity than reference standards. Fitbit-derived PA metrics are not equivalent to existing reference standards. However, they exhibit evidence of construct validity. Consumer-grade fitness trackers such as the Fitbit Inspire HR may therefore be suitable as a PA tracking tool for persons with mild or moderate MS.

## Author summary

Physical activity (PA) is an important aspect of health and well-being. However, PA is often reduced in persons with multiple sclerosis (MS), a neurodegenerative autoimmune disease which affects physical function, motor control, and energy levels. It is of public health interest to increase PA behavior in this population. However, valid and user-friendly methods for tracking PA are required to quantify PA behavior during patients’ daily lives. So-called “research-grade” wearable devices are used for short-term measurements (for example, 7 days), but offer poor user experience and are therefore not suitable for longer-term PA tracking. It is therefore increasingly common for MS researchers to use “consumer-grade” devices such as Fitbits. However, high-quality evidence of their validity in MS populations is limited. In this study, we compared PA metrics derived from a Fitbit device to multiple, validated research-grade methods. While the PA metrics derived from each method were not equivalent, all exhibited the similar evidence of validity. In some cases, Fitbit outperformed research-grade methods. We posit that PA metrics derived from the Fitbit are now suitable for long-term PA tracking in MS populations, and that the resulting longitudinal data has the potential to progress our understanding of world PA behavior in MS populations.

## Introduction

Multiple sclerosis (MS) is a neurodegenerative autoimmune disease which affects physical and cognitive function, motor control, and energy levels. Physical activity (PA) is often reduced in persons with MS (PwMS) [1,2], though it is known to aid in symptom and fatigue management [3–5] and is perceived as an important part of health care by PwMS [6,7]. Managing appropriate amounts of PA is often difficult for PwMS, as overexertion can cause severe short-term fatigue or symptom exacerbations before the benefits of PA are realized [8–10]. To enable the best health outcomes, tools for managing PA and fatigue are desired by PwMS [11].

For such tools to be effective, they must reliably and conveniently track PA over long periods of time, yielding either clinically or personally meaningful information. Consumer-grade PA trackers such as wrist-worn Fitbits are therefore gaining popularity in this population, and have already been used to generate PA outcomes in several large cohort and interventional studies [12–14]. They are easy to use, engaging, inexpensive, and provide meaningful PA metrics which are interpretable within the context of public health guidelines [15]. In addition, these devices enable users to interact with their own data, set goals, and review progress over time. These features promote long-term engagement with remote monitoring technologies [16,17]. The resulting rich, longitudinal data could provide insights into PA behavior not observed in traditional periodic or questionnaire-based PA metrics.

However, only limited evidence of validity is available for any Fitbit device in MS populations. Existing validation studies are primarily conducted in healthy adults, and three recent systematic reviews of such studies cautiously support the validity of Fitbit-derived PA metrics [18–21]. However, validation studies also suggest that these metrics’ accuracies decrease at low activity intensities [20], at slow walking speeds [18,22–24], and with the use of walking aids [25]. Not only do PwMS walk slower healthy controls, they also exhibit different abnormal gait patterns [26,27] and frequently adopt walking aids as their MS progresses [28,29]. It is plausible that these factors affect the validity of Fitbit-derived PA metrics in PwMS. To date, validation studies in PwMS are limited to step count, and do not address the other PA metrics produced by these trackers [30,31]. Given the expanding use of wrist-worn Fitbits to track PA in MS, a thorough evaluation of their validity in this population is warranted.

**Fig 1:**
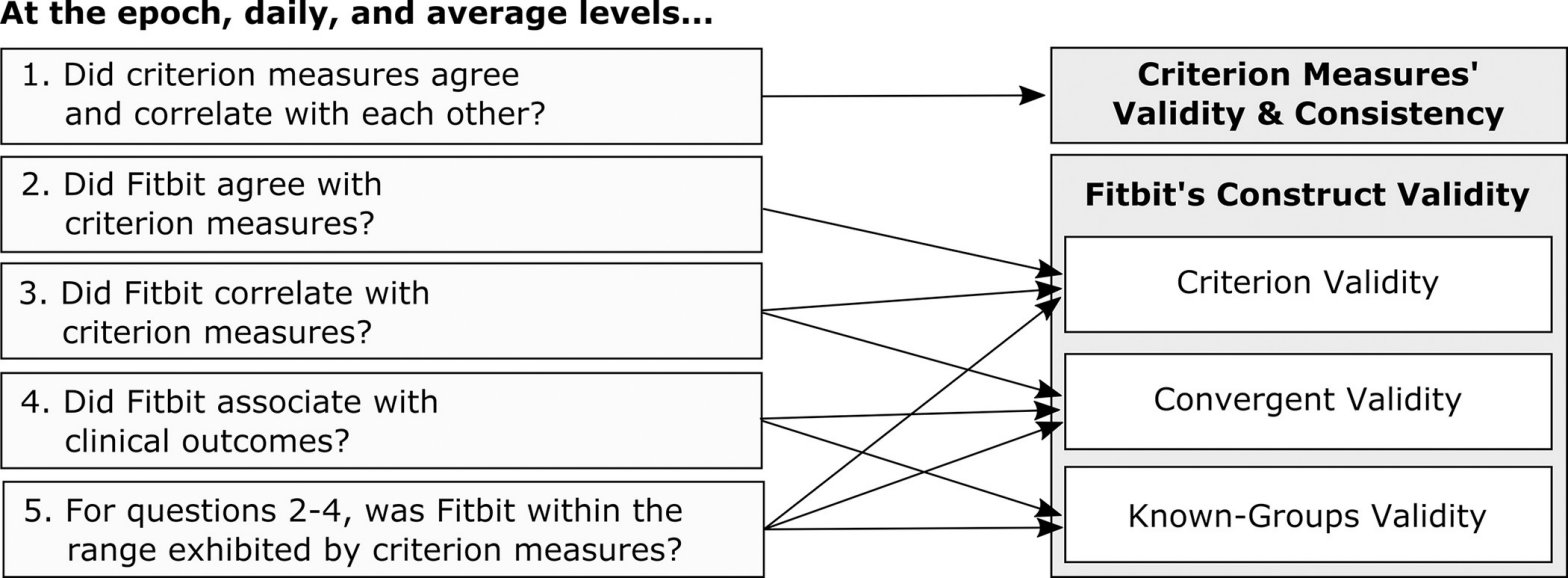
Research questions addressed in this validation study.

In this study, we aimed to expand and update existing evidence on the validity of wrist-worn Fitbit devices in MS populations. We assessed the construct validity of three PA metrics–step count, time spent in PA, and time spent in moderate to vigorous PA (MVPA)–derived from the Fitbit Inspire HR. We did this by comparing Fitbit-derived PA metrics to multiple reference measures (Table 1), and systematically triangulating evidence of their criterion validity, convergent validity, and known-groups validity (Fig 1). This validation study evaluated PA metrics according to validation best practices, accounting for the known shortcomings of existing reference measures [32].

**Table 1:** Methods used to triangulate the validity of Fitbit-derived PA metrics.

Method	Description
***Step count***	
Manual	Scripted tasks were video-recorded and two assessors manually counted steps according to a validated standard operating procedure. The two assessors’ counts were averaged. Manual counts were used as criterion measures during scripted tasks only.
Actigraph (Standard)	During post-processing, a band pass filter is applied to Actigraph’s raw accelerometer signal to remove movement artifact outside the range of human motion. Actigraph’s step count algorithm detects footfalls by identifying peaks in the accelerometer signal, and can therefore be affected by choice of filter. The standard filter was developed by the manufacturer for use in healthy populations, but has also been used in populations with MS. It is known to underestimate step count, especially in populations with walking impairments [33,34].
Actigraph (LFE)	The Low Frequency Extension (LFE) is a revised band pass filter which enhances the Actigraph’s sensitivity to slow movements. It is recommended by the manufacturer in populations with impaired mobility, and is also frequently used in MS populations. The LFE has been shown to increase sensitivity to slow stepping in laboratory settings. However, it is also known to overestimate step count during free-living activity [33].
Fitbit	Fitbit’s proprietary step detection algorithm derived step count from the device’s raw accelerometer signal. Fitbit provides step counts at up to minute-level granularity through its application programming interface (API).
***Time in PA***	
Actigraph (Vertical)	PA intensity is derived from the Actigraph by applying cutpoints to the number of activity counts identified per minute. The Actigraph (Vertical) method differentiates between sedentary behavior and PA depending on whether a minimum cutpoint of 100 vertical axis counts per minute is met [35]. This method was validated on an older model of Actigraph which is roughly equivalent to data derived from the LFE on the Actigraph GT3X. It is widely used in MS populations [36,37].
Actigraph (VM)	The Actigraph Vector Magnitude (VM) method accounts for three-dimensional motion, rather than motion in the vertical axis. This method categorizes minutes as sedentary or PA with a cutpoint of 150 VM counts per minute. It is typically used with the standard filter applied. It was derived from healthy individuals [35,38,39], but is also used in persons with MS [40,41].
Fitbit	Fitbit’s proprietary PA classification algorithm classifies minutes into four intensity categories: sedentary, lightly active, fairly/moderately active, and very active [42]. These categories loosely align with those used by research-grade devices: sedentary, light PA, moderate PA, vigorous PA. We defined all non-sedentary minutes as time in PA. The factors which influence PA classification are not publicly available. However, movement intensity, heart rate, and breathing rate are mentioned by moderators of Fitbit’s support forum [43]. Fitbit provides PA classifications at the minute level through its API, which may then be aggregated into hourly or daily metrics.
***Time in MVPA***	
Actigraph (Uniform)	The Actigraph (Uniform) method was developed to differentiate between light and MVPA in populations with MS and was validated during over-ground walking at multiple speeds. It defines minutes which exceed 1745 vertical axis counts to be MVPA, and uses the LFE filter [44].
Actigraph (Severity)	The Actigraph (Severity) method was developed alongside the Actigraph (Uniform) method, but proposes different cutpoints according to MS severity: • Mild/moderate MS (EDSS < 6.0): 1980 vertical counts per minute • Severe MS (EDSS > = 6.0): 1185 vertical counts per minute Both cutpoints are designed to be used with the LFE filter [44].
Actigraph (Sasaki)	The Actigraph (Sasaki) method uses a cutpoint of 2690 VM counts per minute to differentiate between light and MVPA. This method was developed in healthy controls during treadmill walking at varying speeds [38]. It has since been used in populations with MS [40,45].
Fitbit	We defined all minutes characterized as ‘fairly/moderately active’ and ‘very active’ by Fitbit’s proprietary algorithm as time in MVPA [42]. This grouping aligns with the output of the Actigraph Uniform, Severity, and Sasaki methods, which do not differentiate between moderate PA and vigorous PA [38,44].

MS: multiple sclerosis; PA: physical activity; LFE: Low frequency extension; MVPA: moderate to vigorous physical activity; EDSS: expanded disability status score

## Materials and methods

### Objective

The objective of this study was to assess the construct validity of physical activity (PA) metrics derived from the Fitbit Inspire HR, a consumer-grade fitness tracker. Construct validity is the extent to which an index measure–or the instrument under study–measures the theoretical construct it is supposed to measure [46]. Several sub-types of validity comprise construct validity [47]. In this study, we assess Fitbit-derived PA measures in terms of their criterion validity, known-groups validity, and convergent validity. Criterion validity refers to an instrument’s ability to measure the concept it purports to measure, and is typically assessed through correlations and agreement with a well-validated reference standard, or “criterion measure.” [48] Known-groups validity is the ability of an instrument to discriminate between groups of individuals which are known to differ from each other, such as disease severity strata [49]. Finally, convergent validity refers to a measure’s ability to demonstrate an expected relationship with other theoretically related, clinically relevant constructs [50]. Convergent validity is often assessed through correlation and other association measures.

This validation study was conducted as part of BarKA-MS, a cohort study on the barriers and facilitators to PA in PwMS [51]. It expands upon best practices developed by Johnston et al., [32] who propose a six-step framework for designing and reporting validation studies of consumer wearables: 1) target population, 2) index measure (the measure being validated), 3) testing conditions, 4) criterion measure (the reference standard), 5) data processing methods, and 6) statistical analysis.

### Target population

Our target population was ambulatory PwMS. We recruited a convenience sample of PwMS undergoing elective inpatient neurorehabilitation at the Kliniken Valens between January and November 2021. Participants were eligible if they 1) had a confirmed diagnosis of MS according to their referring physician, 2) were 18 years of age or older, 3) had reduced walking ability but were able to walk independently with or without an assistive device, 4) had access to WiFi and a mobile device in the rehabilitation center and at home, 5) were willing to wear study devices to measure their PA, and 6) were able to answer study questionnaires in German. The BarKA-MS study was composed of two phases (in the clinic and at home). The first phase lasted between one to three weeks depending on the length of the rehabilitation stay and the second phase lasted four weeks. We set a target sample size of 45 participants based on the expected rate of enrollment at Kliniken Valens in the first half of 2021. The recruitment window was then extended due to slower than expected enrollment throughout the COVID pandemic. The ethics committee of the canton of Zurich approved the study protocol (BASEC-no. 2020–02350) and all participants provided written informed consent.

### Index measure

Our index measures–or the measures we aimed to validate–were step count, time in PA, and time in MVPA derived from the Fitbit Inspire HR. The Fitbit Inspire HR is a consumer PA tracker which is worn on the wrist and measures step count, PA intensity, sleep, heart rate, and other fitness metrics at up to minute-level granularity. Participants were given a Fitbit Inspire HR and were instructed to wear it on their non-dominant wrist during the day and if desired at night throughout the course of the study. The accompanying mobile application was installed on each participant’s mobile device, and each participant was given a de-identified, pre-configured study account. Alerts and daily goals were either turned off or set to a minimum, and participants were encouraged to leave these settings off for the duration of the study. Minute-level data were collected and stored through the Fitabase platform (Fitabase, San Diego, California), a cloud-based study management platform which provides industry-standard security measures such as encryption, password protection, access logs, etc. All participants consented to the privacy statements and settings associated with these platforms.

### Testing conditions

According to Johnston et al.’s framework, index measures were compared to criterion measures during laboratory evaluation (i.e., controlled walking tests), semi-free-living evaluation (i.e., scripted assessments which simulate various free-living activities), and free-living evaluation (i.e., during daily living ‘in the wild’) [32]. For brevity, we refer to laboratory evaluations and semi-free living evaluations together as ‘scripted tasks.’

### Laboratory evaluation

Rehabilitation schedule permitting, PA metrics were assessed manually, via the Fitbit, and via criterion measures during a 6-Minute Walk Test [52] in participants’ final week at the clinic. Criterion measures are described in greater detail in the next section. All participants were instructed to cover as much distance in six minutes as possible, and rests were allowed. Participants rested in a seated position for at least three minutes immediately prior to and following the test to allow for confirmation of timestamp alignment between devices.

#### Semi-free-living evaluation

A sub-sample of participants also completed an assessment comprised of five scripted tasks designed to replicate movement patterns regularly encountered in daily life. PA metrics were assessed via the Fitbit and via criterion measures (see below) during these tasks. The semi-free-living evaluation consisted of:

- *Walking with postural transitions*: Participants repeatedly rose from a seated position, walked approximately five meters to an examination bed, lay supine for three seconds, returned to the chair, and sat for three seconds. This task was designed to assess the effect of short walking bouts interrupted with postural transitions.
- *Simulated cleaning*: Participants repeatedly carried a series of glasses, cups, saucers, and towels from one table to another nearby table. During each repetition, participants unfolded and re-folded the towels. This task simulated light PA with short walking bouts in a confined space, frequent direction changes, and weight shifting between legs. We designed this task to simulate working in a kitchen or tidying a room.
- *Sit to stand*: In this task, participants repeatedly rose from and returned to a seated position. This activity further tested how postural transitions are characterized by index and criterion measures.
- *Wheelchair push*: Participants propelled themselves around a circular track in a wheelchair with the Fitbit worn on the outermost wrist to assess how manual wheelchair propulsion, and more generally upper body activity, is characterized.
- *Stair climb and descent*: In this task, participants repeatedly walked up and down two flights of stairs to assess step count accuracy during stair climbing and descent.

These activities were selected and designed in collaboration with subject matter experts at the rehabilitation facility. Each semi-free-living evaluation lasted approximately 30 minutes. Participants were instructed to complete each task at a pace they could maintain safely for three minutes and to use their preferred walking aids. Rests were allowed. Participants rested in a seated position for at least three minutes immediately prior to and following each task to enable confirmation of timestamp alignment and to mitigate fatigue effects.

#### Free-living evaluation

For the purposes of this evaluation, participants wore both the Fitbit and a criterion measure (Actigraph GT3X, see below) under free-living conditions for approximately 14 days. This two-week period was comprised of their final week in the rehabilitation clinic and the following week in their home environment. Participants occasionally wore the devices longer if the rehabilitation period was unexpectedly extended. After participants had worn the device at home for seven days, the participants logged the dates they had worn the devices and returned the Actigraph GT3X to investigators by mail. Participants continued to wear the Fitbit as part of the BarKA cohort study.

### Criterion measures

Average manual step counts were considered the criterion measure for assessing Fitbit’s step count algorithm during scripted tasks. Tasks were video-recorded and two assessors manually counted steps according to a validated standard operating procedure (S1 Text).

Several additional criterion measures were derived from the Actigraph GT3X (Manufacturing Technology, Inc., FL, USA), a research-grade accelerometer which has been validated in PwMS [53,54]. Actigraph devices were initialized in Actilife 6.0 with a sampling rate of 30Hz and worn on the right hip. However, multiple data processing methods exist to derive PA metrics in this population (Table 1) [38,44,55]. These methods use different data (i.e., 1-dimensional vs. 3-dimensional movement) and processing methods (i.e., standard vs. highly sensitive filtering) to calculate PA metrics. However, the Fitbit is not expected to agree exactly with any of the criterion measures derived from the Actigraph GT3X (Table 1). The Actigraph measures were derived and validated for wear on the hip [35,38,44], whereas Fitbit is wrist-worn. The Actigraph GT3X-based methods derive PA metrics from an accelerometer only [35,38,44]. The factors which influence PA classification are not publicly available, though support resources suggest that movement intensity, heart rate, and breathing rate may influence PA estimation [43]. Finally, Actigraph-derived measures are non-equivalent with each other [56]. Any Actigraph method may therefore impart criterion standard bias if compared to Fitbit as a single criterion measure [57].

We therefore opted to assess the metrics derived from Fitbit through triangulation [58] in an agreement validation study [57] and through an assessment of construct validity. Criterion measures for step count, time in PA, and time in MVPA were derived from Actigraph through multiple established methods (Table 1). Two Actigraph-based methods were used to derive step count (referred to as Actigraph (Standard) and Actigraph (LFE)) [59], two methods were used to derive time in PA (Actigraph (Vert) [35] and Actigraph (VM)) [38], and three methods were used to derive time in MVPA (Actigraph (Uniform), [44] Actigraph (Severity), [44] and Actigraph (Sasaki)) [38].

Construct validity was further evaluated by quantifying the relationship between PA metrics and theoretically-related clinical assessments. Convergent validity was assessed through associations with patient reported outcomes and clinical outcome measures. Patient reported outcomes included the MS Walking Scale– 12 (MSWS-12), a patient-reported measure of walking ability and its impact on daily activities [60,61] and the International PA Questionnaire (IPAQ), a self-assessment of PA during the previous seven days [62]. Clinical measures included the Expanded Disability Status Scale (EDSS) [63]; the 10-meter Gait Speed test (10mGS) [64]; and the 6-Minute Walk Test (6MWT) [65]. These measures were assessed during the last week of rehab, except for the IPAQ, which was reported by participants following the free-living assessment. Known-groups validity was assessed by comparing PA metrics between subgroups according to established cutoffs of clinical scales. Disease severity strata were defined as mild (EDSS < 4.0), moderate (EDSS 4.0–5.5), and severe (EDSS 6.0–6.5) body function impairment, consistent with previous studies [44].

### Data processing

Actigraph data were uploaded to Actilife, filtered to remove non-human movement artifact with both the standard filter and the low frequency extension (LFE), aggregated into one minute epochs, and exported for further processing. Step count, PA intensity (sedentary behavior, LPA, MVPA), and heart rate data derived from the Fitbit Inspire HR were calculated according to Fitbit’s proprietary algorithms and extracted in one minute epochs. All processing was conducted in R, version 4.1.0 in the RStudio environment, version 1.4.1717. Validated algorithms (Table 1) were applied to derive PA intensity and step count.

Non-wear time was defined as 30 minutes of continuous zeros with a 2-minute spike tolerance [66]. For Actigraph, this definition referred to epochs with zeroes in the x, y and z axes, and for Fitbit this referred to epochs with zero step count, sedentary PA categorization, and no registered heart rate. Wear periods shorter than 10 minutes were discarded to reduce false positives in wear time resulting from short spikes. Days with at least 10 hours of wear time during waking hours were considered valid [67], and participants with at least two valid days were included in this analysis [68]. Epochs in which both devices were worn during waking hours (6AM to 11PM) on valid days were included in aggregation and analysis. Data categorized as non-wear time and epochs which occurred on non-valid days were removed. The day participants left the clinic and traveled home was not included in this analysis, as these days did not represent ‘normal’ activity. To limit the effects of differential wear patterns on agreement analyses, only minutes during which both the Fitbit and the Actigraph were worn were included in data aggregation and further analysis.

#### Data aggregation

For each method, PA data were then aggregated into three levels of granularity for agreement analysis: “epoch-level”, “daily”, and “average” PA. Epoch-level data was used to evaluate absolute agreement between PA metrics over short periods of time and during diverse activities of daily living. Timestamp alignment within one minute was confirmed according to visit notes, videos, and manual inspection for each participant. Minute-level step counts were aggregated into 5 minute epochs. An agreement window of plus or minus one minute was applied in a pairwise fashion to minute-level PA intensity metrics. This window accounted for the effects of timestamp misalignment and the potential dependency of Fitbit’s PA algorithm on heart rate. An epoch was considered in agreement if Fitbit-derived PA intensity yielded the same categorization as Actigraph-derived PA intensity within a window of plus or minus one minute of the Actigraph’s timestamp. Daily PA metrics were calculated by summing all included minute-level data per patient per day. Days in both the rehab setting and the home setting were included in analyses at the daily level of aggregation. Average-level PA metrics were calculated for the home environment only by averaging each participant’s daily PA metrics over all valid days, consistent with previous PA study outcomes in MS populations [40,69].

#### Data labeling

Data collected during laboratory and semi-free-living evaluations were extracted and labeled by consulting visit notes, video timestamps, and manual inspection. Manual and device-derived step counts were calculated for each scripted task and for the rests between tasks.

### Statistical analysis

Agreement of categorical data was assessed through a multi-level implementation of Fleiss’ kappa assuming participant-level random effects [70]. Differences in PA categorizations during individual scripted tasks were identified through Fisher exact tests. Kruskal-Wallis tests, Wilcoxon signed-rank tests, Pearson product-moment correlation coefficients (Pearson’s r), Lin’s concordance correlation coefficients (CCC) [71] evaluated the differences, correlations, and absolute agreement between measures for continuous and count data. Bland Altman plots [72] visualized the mean bias and limits of agreement at the daily level. At the epoch and daily level, Pearson’s r, CCC, and Bland Altman statistics were adjusted for patient-level random effects according to the procedures defined by Parker et al. [73] Pearson’s r was selected because it was adjustable for patient-level random effects, and data were visually assessed for approximate normal distributions. Confidence intervals were derived through bootstrapping. In sensitivity analysis, these analyses were repeated for each disease severity stratum. For data collected during scripted tasks, this analysis was conducted for all scripted tasks together, accounting for task-level random effects as described by Parker et al. [73] Wilcoxon-Mann-Whitney tests and Wilcoxon effect sizes [74] quantified the existence and magnitude of differences across known groups. Pearson’s r quantified the relationships between average PA metrics and clinical measures.

### Triangulation

“Triangulation” refers to the use of more than one approach to address a research question. By combining multiple methods and comparing results from different perspectives, the limitations of each method individually can be contextualized and addressed [58]. We developed a qualitative triangulation process to assess Fitbit’s construct validity relative to several criterion measures at several levels of data aggregation. We did this by qualitatively considering the devices’ performance during each evaluation according to a pre-defined list of considerations (Fig 1).

Each of these research questions was addressed at five levels of data aggregation: during scripted tasks, at the epoch, daily, and average level, and across the three disease severity strata. Analyses were rated according to the level of agreement and correlation exhibited by the various PA metrics. Categories aligned with widely accepted, though arbitrary, interpretations of correlations and kappa statistics found in the literature [75,76]:

- ++: Excellent agreement or strong correlations (0.75–1.0)
- +: Fair to good agreement or moderate correlations (0.4–0.74)
-: Poor agreement, weak correlations (0.2–0.39)
--: Very weak or complete lack of agreement or correlation (<0.2)
- ++/+/-/--: Evidence was mixed

## Results

### Participant characteristics

**Fig 2:**
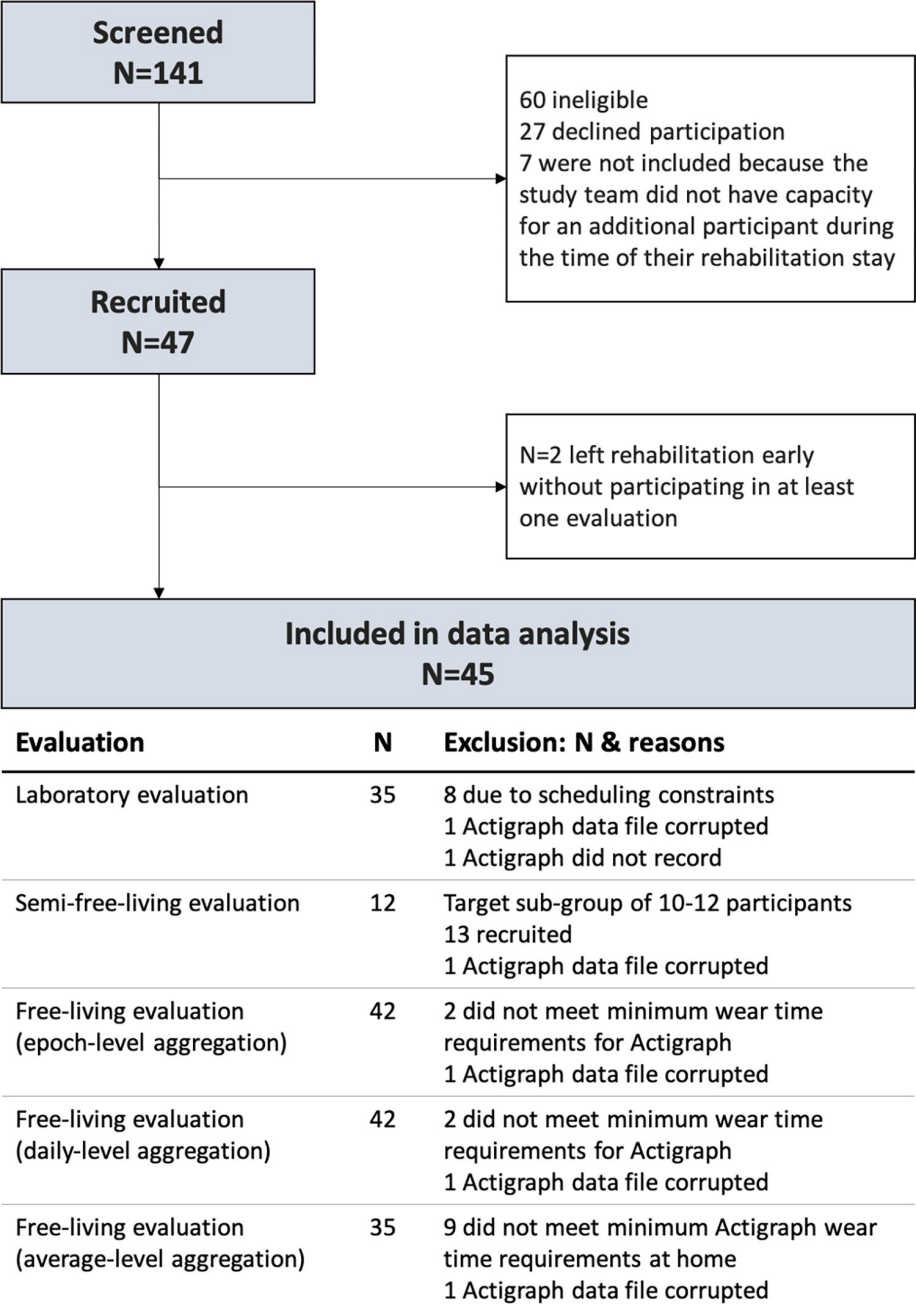
Flowchart of recruitment and participation in this study.

Of the 47 participants originally enrolled, two participants left rehabilitation early and had to be excluded from the study. Of the 45 remaining participants, 29 (64.4%) were female and 19 (35.6%) were male. The median age was 46 (IQR: 40–51) years. Median EDSS was 4.0 (Range: 2.0–6.5), indicating moderate disease severity of the population. Most participants had either secondary-progressive MS (42.2%) or relapsing-remitting MS (40%). The median time from diagnosis was 11 years (IQR 5–21). The participants who completed the 6MWT varied in average walking cadence (Median (Range): 109 (61–146) steps per minute) and walking aid use (none: 23, walking sticks: 10, rollator: 2). Due to rehabilitation schedules, Actigraph wear compliance, and Actigraph data corruption, not all participants were included in all evaluations. The number of participants included in each analysis are described in Fig 2, and their characteristics are described in S1 Table.

During free-living evaluations, participants wore Fitbits for an average of 16.4 (Standard deviation: 0.9) hours on 12.9 (1.9) valid days, whereas they wore the Actigraph for an average of 12.1 (0.9) hours on 8.6 (3.2) valid days.

### Step count

#### 1. Step count: Did criterion measures agree and correlate with each other?

**Fig 3:**
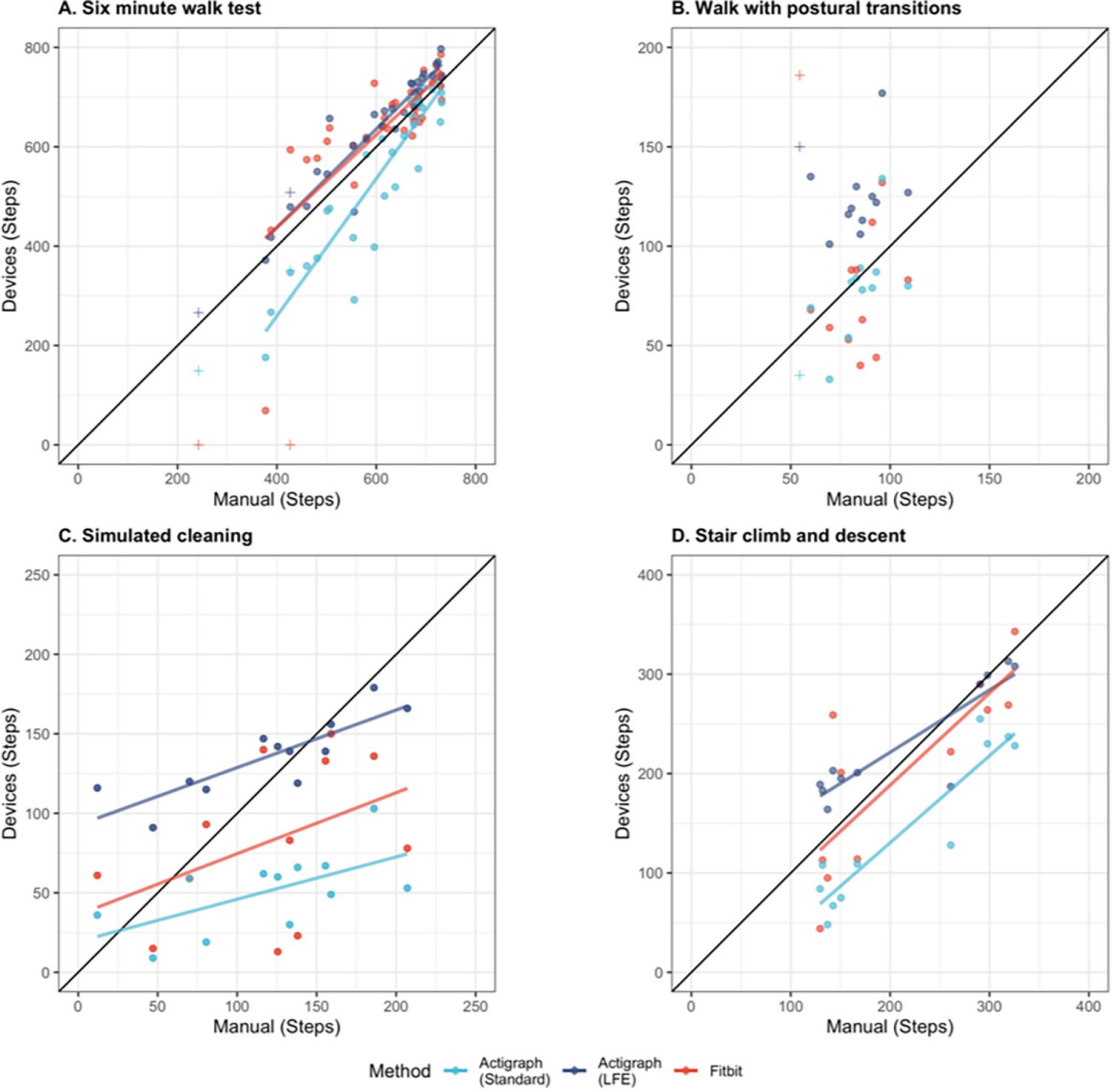
Manual and device-derived step counts during scripted walking tasks. Walking was assessed A) during a six-minute walk test, B) during walking interspersed with postural transitions, C) during a simulated cleaning task, and D) during stair climbing and descent. Measurements in perfect agreement would fall along the indicated diagonal black line. Regression lines (A, C, D) demonstrate deviations between actual and perfect agreement. Regression lines were omitted from B because the population did not exhibit sufficient variation in step count to yield linear trends. Fitbit registered no steps for two patients who used rollators (noted with +) during the 6-minute walk test (A). One participant’s dyskinesia (noted with +) caused Fitbit to overestimate step count while walking with postural transitions (B), though not on other tasks when she could use a rollator or hold on to nearby structures for balance.

**Fig 4:**
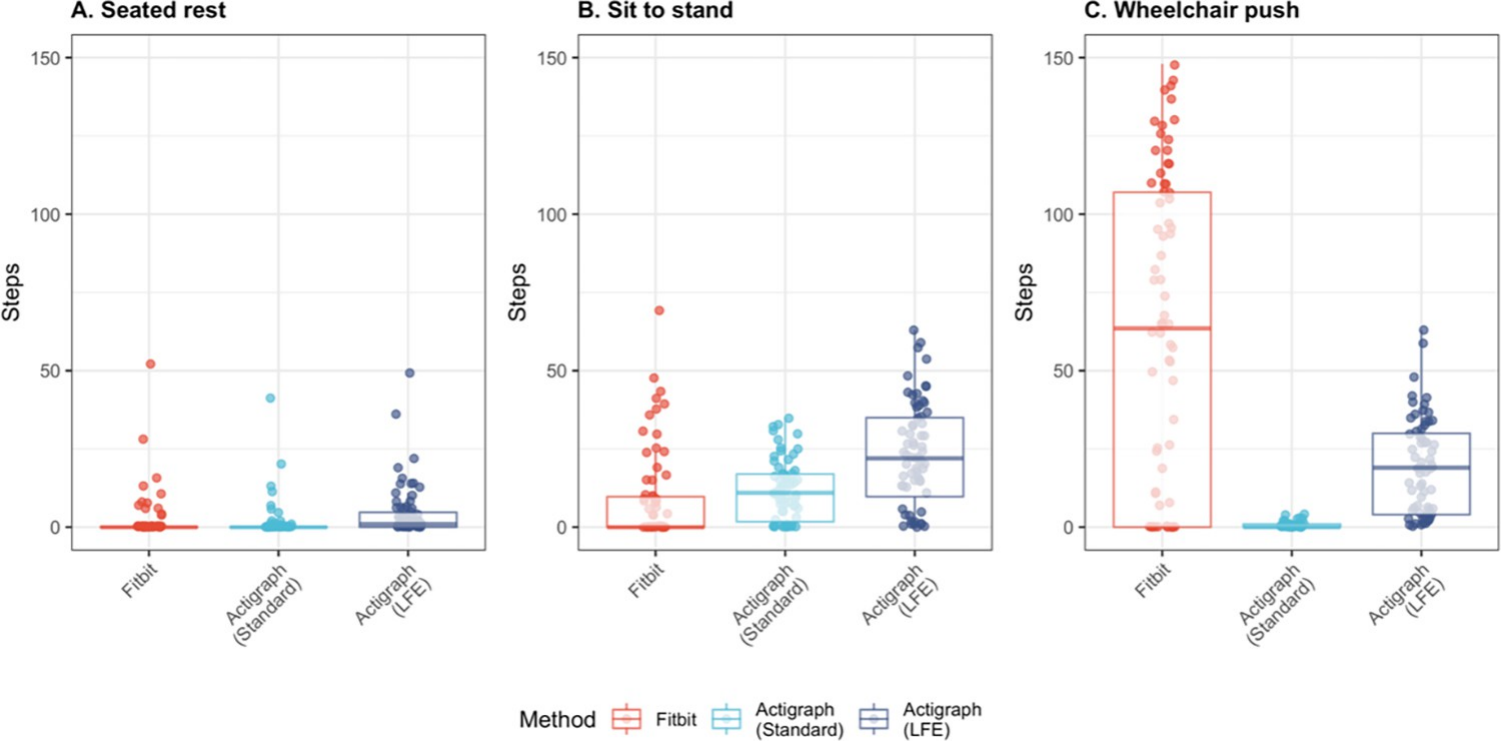
Steps per minute derived from Fitbit and Actigraph during scripted non-walking tasks. Step counts were generated by each method–Fitbit, Actigraph (Standard), and Actigraph (LFE)–during tasks in which no manual steps were identified: A) Seated at rest, B) during the sit to stand task, and C) while pushing a wheelchair.

During scripted tasks, both Actigraph methods–Actigraph (Standard) and Actigraph (LFE)–demonstrated strong correlation and good agreement with manual counts (r: 0.97–0.98; CCC: 0.68–0.73) and with each other (r: 0.97; CCC: 0.60) (Table 2). Actigraph (Standard) often underestimated step count compared to manual counts, and Actigraph (LFE) often mischaracterized non-walking movement and postural transitions as steps (Fig 3 and Fig 4).

**Table 2:** Correlation and agreement between step counts derived from Fitbit and Actigraph.

Comparison	r [95% CI]	CCC [95% CI]
***Scripted tasks***		
Manual vs Act(Stand)^a^	0.97 [0.77–0.99]	0.68 [0.37–0.82]
Manual vs Act(LFE)^a^	0.98 [0.78–0.99]	0.73 [0.13–0.84]
Act(Stand) vs Act(LFE)^a^	0.97 [0.85–0.99]	0.60 [0.06–0.76]
Fitbit vs. Manual	0.92 [0.63–0.98]	0.66 [0.14–0.80]
Fitbit vs. Act(Stand)	0.92 [0.65–0.97]	0.55 [0.08–0.72]
Fitbit vs. Act(LFE)	0.93 [0.65–0.98]	0.65 [0.24–0.77]
***Free living*, *Epoch level***		
Act(Stand) vs Act(LFE) ^a^	0.27 [0.24–0.30]	0.03 [0.02–0.04]
Fitbit vs. Act(Stand)	0.22 [0.19–0.25]	0.04 [0.03–0.05]
Fitbit vs. Act(LFE)	0.22 [0.19–0.25]	0.03 [0.02–0.05]
***Free living*, *Daily level***		
Act(Stand) vs Act(LFE) ^a^	0.88 [0.84–0.91]	0.15 [0.11–0.20]
Fitbit vs. Act(LFE)	0.82 [0.78–0.87]	0.33 [0.22–0.43]
Fitbit vs. Act(Stand)	0.80 [0.75–0.85]	0.44 [0.32–0.57]
***Free living*, *Average level***		
Act(Stand) vs Act(LFE) ^a^	0.89 [0.78–0.94]	0.27 [0.16–0.37]
Fitbit vs. Act(LFE)	0.86 [0.74–0.93]	0.50 [0.34–0.63]
Fitbit vs. Act(Stand)	0.82 [0.67–0.90]	0.65 [0.47–0.77]

^a^ Comparison between two criterion measures

Act: Actigraph; Stand: Standard; LFE: Low frequency extension; r: Pearson correlation coefficient; CI: confidence interval; CCC: Lin’s Concordance correlation coefficient

During free-living evaluation, the two methods exhibited weak correlation and no agreement at the epoch level (r: 0.27; CCC: 0.03) and strong correlation but poor agreement at the daily (r: 0.88; CCC: 0.15) and average levels (r: 0.89; CCC: 0.27) (Table 2). These patterns were consistent across disease severity strata. (S2 Table)

#### 2. Step count: Did Fitbit agree with criterion measures?

During scripted tasks, Fitbit-derived step counts demonstrated good agreement with manual counts (CCC: 0.66) and with Actigraph-derived counts (CCC (Standard): 0.55, CCC(LFE): 0.65) (Table 2). During scripted walking tasks, Fitbit-derived step counts were consistent with manual and Actigraph-derived counts, with one exception: Fitbit registered zero steps for two participants who used rollators during the test. Walking stick use did not appear to effect step count during the 6MWT or other walking tasks. Upper-body movement was often mischaracterized as steps by Fitbit (Fig 4).

**Fig 5:**
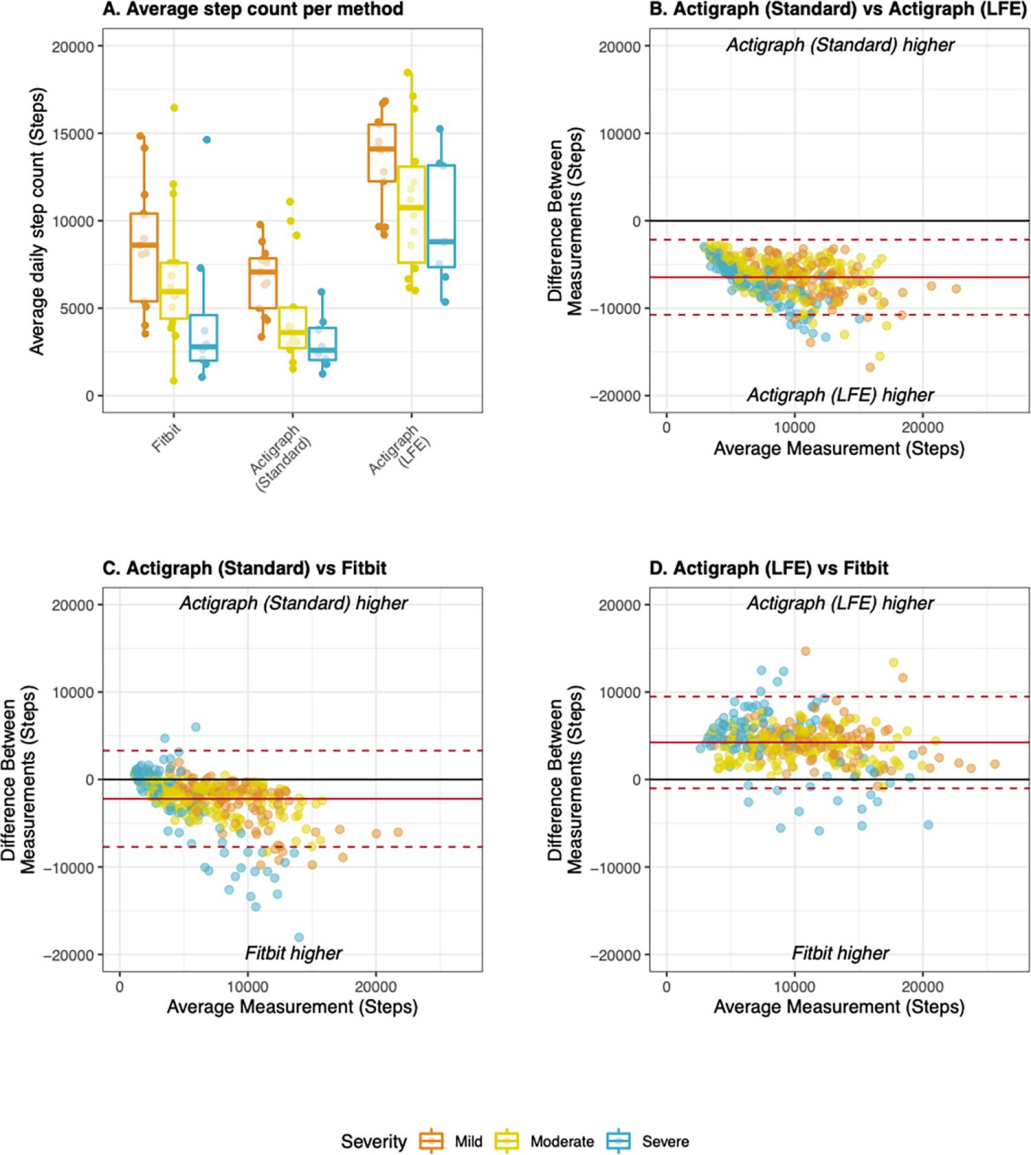
Step counts derived from Fitbit and Actigraph during free-living evaluation. A) Average step counts derived from the Fitbit, Actigraph (Standard), and Actigraph (LFE) stratified by disease severity. B) Bland Altman plot comparing Actigraph methods to each other; C) Bland Altman plot comparing Fitbit to Actigraph (Standard), D) Bland Altman plot comparing Fitbit to Actigraph (LFE). All Bland Altman plots display data collected during rehab and at home at the daily level of aggregation. Mean bias (solid line) and limits of agreement (dotted lines) were adjusted for patient-level random effects. Mild MS: EDSS < 4.0, Moderate MS: EDSS 4.0–5.5, Severe MS: EDSS 6.0–6.5.

During free-living evaluation, agreement between Fitbit and Actigraph was substantially reduced compared to scripted tasks. However, step counts derived from the Fitbit consistently exhibited equivalent or higher agreement with each of the Actigraph methods than the Actigraph methods did with each other (Table 2). Fitbit produced significantly higher step counts than Actigraph (Standard) and significantly lower counts than Actigraph (LFE) (both p < 0.001) (Fig 5A). At the epoch level, Fitbit exhibited no agreement with either Actigraph method (Table 2). Between two and four percent of epochs exhibited substantial inconsistency, with minimal steps detected by one device and large steps counts detected by the other (S1 Fig). Fitbit demonstrated poor to good agreement with each Actigraph method at the daily (CCC: 0.33–0.44) and average levels (CCC: 0.50–0.65) (Table 2). Bland Altman analysis yielded wide limits of agreement, further confirming the weak agreement of daily step counts (Fig 5B–5D). Agreement tended to be highest for participants with moderate MS and lowest for participants with severe MS (S2 Table).

#### 3. Step count: Did Fitbit correlate with criterion measures?

During scripted tasks, Fitbit-derived step counts were strongly correlated with those derived from criterion measures (r (Standard): 0.92, r(LFE): 0.93) (Table 2).

During free-living evaluation, Fitbit and Actigraph-derived step count metrics were weakly correlated at the epoch level (r: 0.22), but strongly correlated at the daily (r: 0.80–0.82) and average levels (r: 0.76–0.86) (Table 2). At all levels of aggregation, correlations between Fitbit and Actigraph-derived step counts were similar in magnitude to that of the two Actigraph methods (Table 2). Trends were consistent across disease severity strata (S2 Table).

#### 4. Step count: Did Fitbit associate with clinical outcomes?

Average step counts derived from the Fitbit during free-living evaluation exhibited moderate to strong correlations with most clinical measures (Table 3). These associations were similar in magnitude to those exhibited by Actigraph. Fitbit-derived step count also demonstrated the expected differences between subgroups, though effect sizes for Fitbit were lower than Actigraph-derived counts (Table 3).

**Table 3:** Correlations between device-derived physical activity metrics and clinical measures.

	MSWS-12	IPAQ	EDSS	6MWT	10mGS	Mild vs Moderate/Severe (Based on EDSS)	
	r [95% CI]	r [95% CI]	r [95% CI]	r [95% CI]	r [95% CI]	p	ES [95% CI]
***Step count***							
Fitbit	-0.52 [-0.73 - -0.22]	0.92 [0.66–0.98]	-0.50 [-0.72 - -0.20]	0.62 [0.31–0.81]	-0.47 [-0.70 - -0.16]	0.0358	0.36 [0.05–0.62]
Act (Stand)	-0.67 [-0.82 - -0.43]	0.90 [0.57–0.98]	-0.64 [-0.80 - -0.39]	0.72 [0.47–0.86]	-0.65 [-0.81 - -0.40]	0.0022	0.52 [0.23–0.75]
Act (LFE)	-0.56 [-0.76 - -0.28]	0.76 [0.20–0.95]	-0.48 [-0.70 - -0.17]	0.58 [0.25–0.78]	-0.61 [-0.78 - -0.34]	0.0194	0.40 [0.11–0.65]
***Physical activity***							
Fitbit	-0.51 [-0.72 - -0.20]	0.93 [0.71–0.99]	-0.48 [-0.70 - -0.18]	0.52 [0.17–0.75]	-0.40 [-0.65 - -0.07]	0.0876	0.30 [0.02–0.57]
Act (Vert)	-0.45 [-0.69 - -0.14]	0.77 [0.21–0.95]	*-0*.*27 [-0*.*55–0*.*07]*	*0*.*36 [-0*.*02–0*.*65]*	-0.43 [-0.67 - -0.11]	0.0942	0.29 [0.02–0.59]
Act (VM)	-0.42 [-0.66 - -0.09]	0.69 [0.05–0.93]	*-0*.*26 [-0*.*55–0*.*08]*	*0*.*28 [-0*.*11–0*.*60]*	-0.40 [-0.65 - -0.08]	0.1521	0.25 [0.01–0.55]
***Moderate to vigorous physical activity***							
Fitbit	-0.42 [-0.66 - -0.09]	0.83 [0.36–0.96]	-0.37 [-0.63 - -0.05]	0.54 [0.20–0.76]	-0.45 [-0.68 - -0.13]	0.0508	0.34 [0.04–0.63]
Act (Uni)	-0.56 [-0.76 - -0.28]	*0*.*62 [-0*.*07–0*.*91]*	-0.51 [-0.72 - -0.21]	0.63 [0.33–0.82]	-0.51 [-0.72 - -0.20]	0.0004	0.60 [0.34–0.79]
Act (Sev)	-0.46 [-0.69 - -0.14]	*0*.*49 [-0*.*26–0*.*87]*	*-0*.*23 [-0*.*52–0*.*11]*	0.51 [0.15–0.74]	*-0*.*31 [-0*.*59–0*.*03]*	0.0097	0.45 [0.15–0.68]
Act (Sasaki)	*-0*.*32 [-0*.*59–0*.*02]*	*0*.*26 [-0*.*49–0*.*79]*	-0.35 [-0.62 - -0.02]	*0*.*38 [0*.*00–0*.*66]*	*-0*.*33 [-0*.*60–0*.*01]*	0.0284	0.38 [0.08–0.63]

Point estimates which did not reach statistical significance, defined here as the 95% confidence intervals excluding 0, are shown in italics.

Act: Actigraph; Stand: Standard; LFE: Low frequency extension; Vert: Vertical; VM: Vector Magnitude; Uni: Uniform; Sev: Severity; ES: effect size; r: Pearson’s correlation coefficient

### Time in physical activity

#### 1. Time in PA: Did criterion measures agree and correlate with each other?

During scripted tasks, the two Actigraph methods–Actigraph (Vertical) and Actigraph (VM)–exhibited excellent epoch-level agreement (Fleiss’ k: 0.93) (Table 4). During free-living evaluation, epoch-level agreement decreased slightly, but remained high (k: 0.75). At the daily level, the two methods exhibited strong correlation (r: 0.78) but poor agreement (CCC: 0.34), though agreement increased when time in PA was averaged across all valid days (r: 0.92, CCC: 0.71) (Table 4). Trends were consistent across disease severity strata (S3 Table).

**Table 4:** Correlation and agreement between time in physical activity and moderate to vigorous physical activity derived from Fitbit and Actigraph.

	Epoch level, Scripted tasks	Epoch level, Free living	Daily level, Free living		Average level, Free living	
Comparison	k [95% CI]	k [95% CI]	r [95% CI]	CCC [95% CI]	r [95% CI]	CCC [95% CI]
***Physical Activity***						
Act(VM) vs Act(Vert) ^a^	0.93 [0.88–0.97]	0.75 [0.73–0.77]	0.78 [0.74–0.83]	0.34 [0.25–0.41]	0.92 [0.85–0.96]	0.71 [0.57–0.81]
Fitbit vs Act(Vert)	0.87 [0.72–1.02]	0.76 [0.73–0.80]	0.74 [0.67–0.80]	0.36 [0.21–0.50]	0.72 [0.50–0.85]	0.52 [0.32–0.68]
Fitbit vs Act(VM)	0.85 [0.71–0.99]	0.62 [0.58–0.66]	0.82 [0.77–0.86]	0.18 [0.11–0.26]	0.76 [0.57–0.87]	0.35 [0.20–0.49]
***Moderate to Vigorous Physical Activity***						
Act(Uni) vs Act(Sev) ^a^	0.84 [0.77–0.91]	0.82 [0.75–0.89]	0.79 [0.68–0.86]	0.63 [0.46–0.74]	0.89 [0.79–0.94]	0.87 [0.77–0.93]
Act(Uni) vs Act(Sasaki) ^a^	0.79 [0.63–0.94]	0.73 [0.66–0.80]	0.82 [0.72–0.88]	0.63 [0.47–0.73]	0.90 [0.82–0.95]	0.88 [0.78–0.94]
Act(Sev) vs Act(Sasaki) ^a^	0.76 [0.63–0.88]	0.68 [0.60–0.76]	0.78 [0.65–0.85]	0.56 [0.38–0.66]	0.76 [0.57–0.87]	0.72 [0.53–0.84]
Fitbit vs Act(Uni)	*-0*.*18 [-0*.*48–0*.*13]*	0.39 [0.27–0.51]	0.68 [0.56–0.80]	0.45 [0.27–0.63]	0.80 [0.64–0.90]	0.80 [0.64–0.89]
Fitbit vs Act(Sev)	*-0*.*18 [-0*.*48–0*.*13]*	0.42 [0.31–0.54]	0.65 [0.55–0.75]	0.41 [0.27–0.56]	0.64 [0.40–0.81]	0.63 [0.39–0.79]
Fitbit vs Act(Sasaki)	*-0*.*18 [-0*.*46–0*.*10]*	0.41 [0.31–0.52]	0.67 [0.54–0.79]	0.44 [0.27–0.58]	0.79 [0.62–0.89]	0.78 [0.61–0.88]

^a^ Comparison between two criterion measures

Point estimates which did not reach statistical significance, defined here as the 95% confidence intervals excluding 0, are shown in italics.

Act: Actigraph; Vert: Vertical; VM: Vector Magnitude; Uni: Uniform; Sev: Severity; k: Fleiss’ kappa; CI: confidence interval; r: Pearson correlation coefficient; CCC: Lin’s Concordance correlation coefficient

#### 2. Time in PA: Did Fitbit agree with criterion measures?

**Fig 6:**
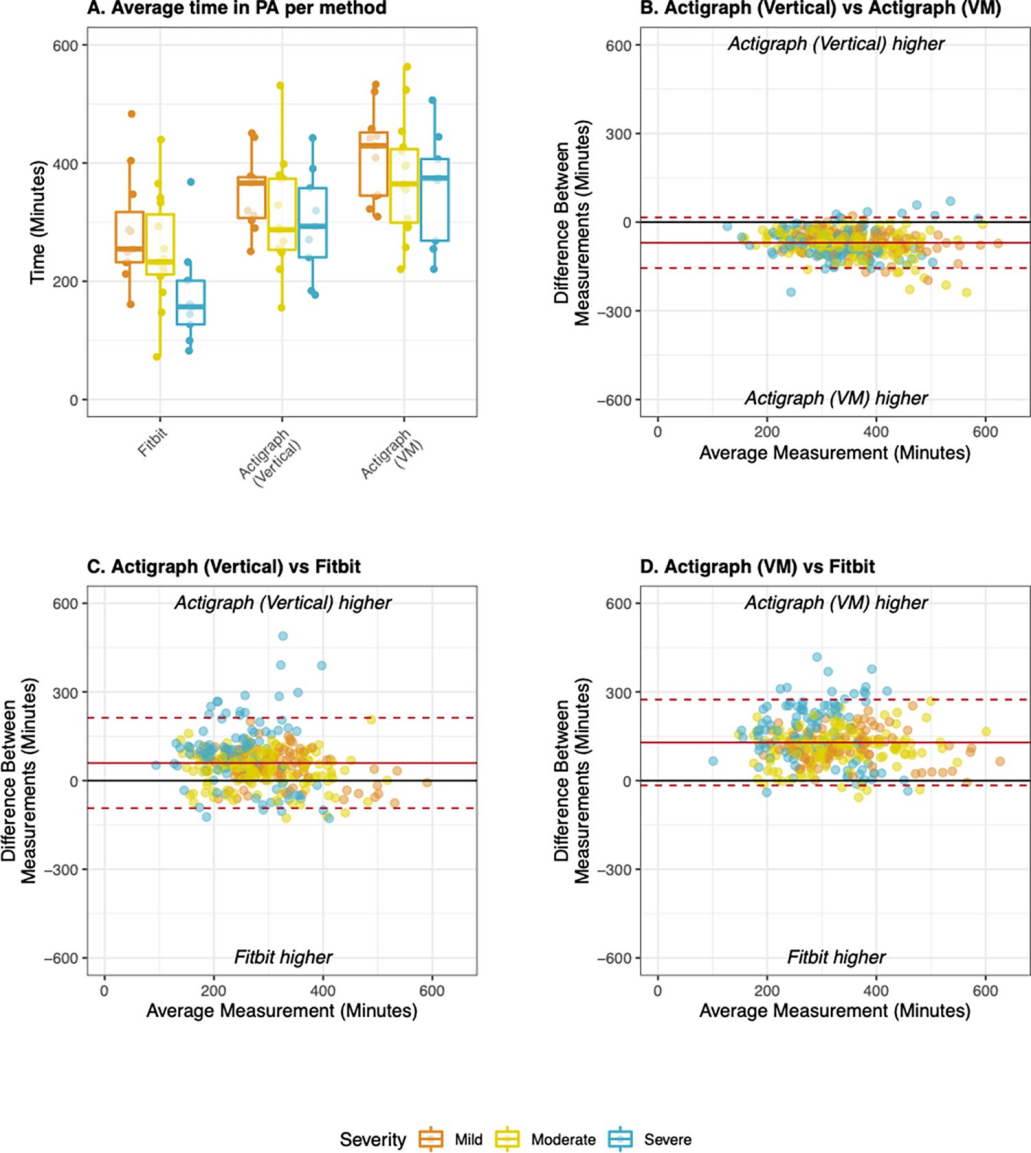
Total time in physical activity derived from Fitbit and Actigraph during free-living evaluation. A) Average time in PA derived from the Fitbit, Actigraph (Vertical), and Actigraph (VM) stratified by disease severity; B) Bland Altman plot comparing both Actigraph methods to each other; C) Bland Altman plot comparing Fitbit to Actigraph (Vertical), D) Bland Altman plot comparing Fitbit to Actigraph (VM). All Bland Altman plots display data collected during rehab and at home at the daily level of aggregation. Mean bias (solid line) and limits of agreement (dotted lines) were adjusted for patient-level random effects. Mild MS: EDSS < 4.0, Moderate MS: EDSS 4.0–5.5, Severe MS: EDSS 6.0–6.5.

During scripted tasks, epoch-level agreement between Fitbit, manual counts, and the two Actigraph methods was excellent (k: 0.85–0.87) (Table 4). During free-living evaluation, agreement was moderate to excellent at the epoch level (k: 0.62–0.76), poor at the daily level (CCC: 0.18–0.36) and weak to moderate at the average level (CCC:0.35–0.52). Fitbit consistently exhibited higher agreement with the Actigraph (Vertical) method than the Actigraph (VM) method (Table 4). However, both Actigraph methods registered significantly more time in PA than Fitbit during free-living PA and limits of agreement on Bland Altman plots were wide for all pairs of measures (Fig 6). Agreement was consistent across subgroups with mild and moderate MS, but was consistently lower in the subgroup with severe MS (S3 Table).

#### 3. Time in PA: Did Fitbit correlate with criterion measures?

During free-living evaluation, correlations between Fitbit and Actigraph-derived time in PA were moderate to strong at the daily (r: 0.74–0.82) and average (r: 0.72–0.76) levels (Table 4). Correlations at the daily level were consistent across subgroups (r: 0.70–0.84), but those at the average level were lower in the subgroup with severe MS (Mild–r: 0.87–0.89; Moderate–r: 0.83–0.87; Severe–r:0.38–0.48)(S3 Table).

#### 4. Time in PA: Did Fitbit associate with clinical outcomes?

Fitbit consistently exhibited moderate to strong correlations with clinical outcome measures, though no method exhibited differences between mild and moderate or severe MS (Table 3). These relationships were either similar to or stronger than those exhibited by Actigraph-derived PA metrics.

### Time in moderate to vigorous physical activity

#### 1. Time in MVPA: Did criterion measures agree and correlate with each other?

The three Actigraph methods–Actigraph (Uniform), Actigraph (Severity), and Actigraph (Sasaki)–exhibited excellent pairwise agreement at the epoch level during scripted tasks (k: 0.76–0.84) and good to excellent agreement during free-living evaluation (k: 0.68–0.82) (Table 4). At the daily level, the three methods exhibited strong pairwise correlations (r: 0.78–0.82) and good pairwise agreement (CCC: 0.56–0.63) (Table 4). Correlations and agreement further increased when time in PA was averaged across all valid days (r: 0.76–0.90, CCC: 0.72–0.88) (Table 4).

Trends were not consistent across disease severity strata. Correlation and agreement between Actigraph methods were consistently lower in persons with severe MS compared to those with mild or moderate MS. Correlation and agreement also tended to be higher in the subgroup with moderate MS compared to that with mild MS, though this was not consistent across all levels of data aggregation. (S4 Table).

#### 2. MVPA: Did Fitbit agree with criterion measures?

During scripted tasks, Fitbit and Actigraph exhibited no agreement in their categorization of MVPA (Table 4). The Fitbit rarely classified any scripted task as MVPA, whereas Actigraph methods frequently classified the 6MWT, Sit to Stand, Stair Climbing, and Walking with Postural Transitions tasks as MVPA. It remains unclear which activities register as MVPA on the Fitbit in this population.

**Fig 7:**
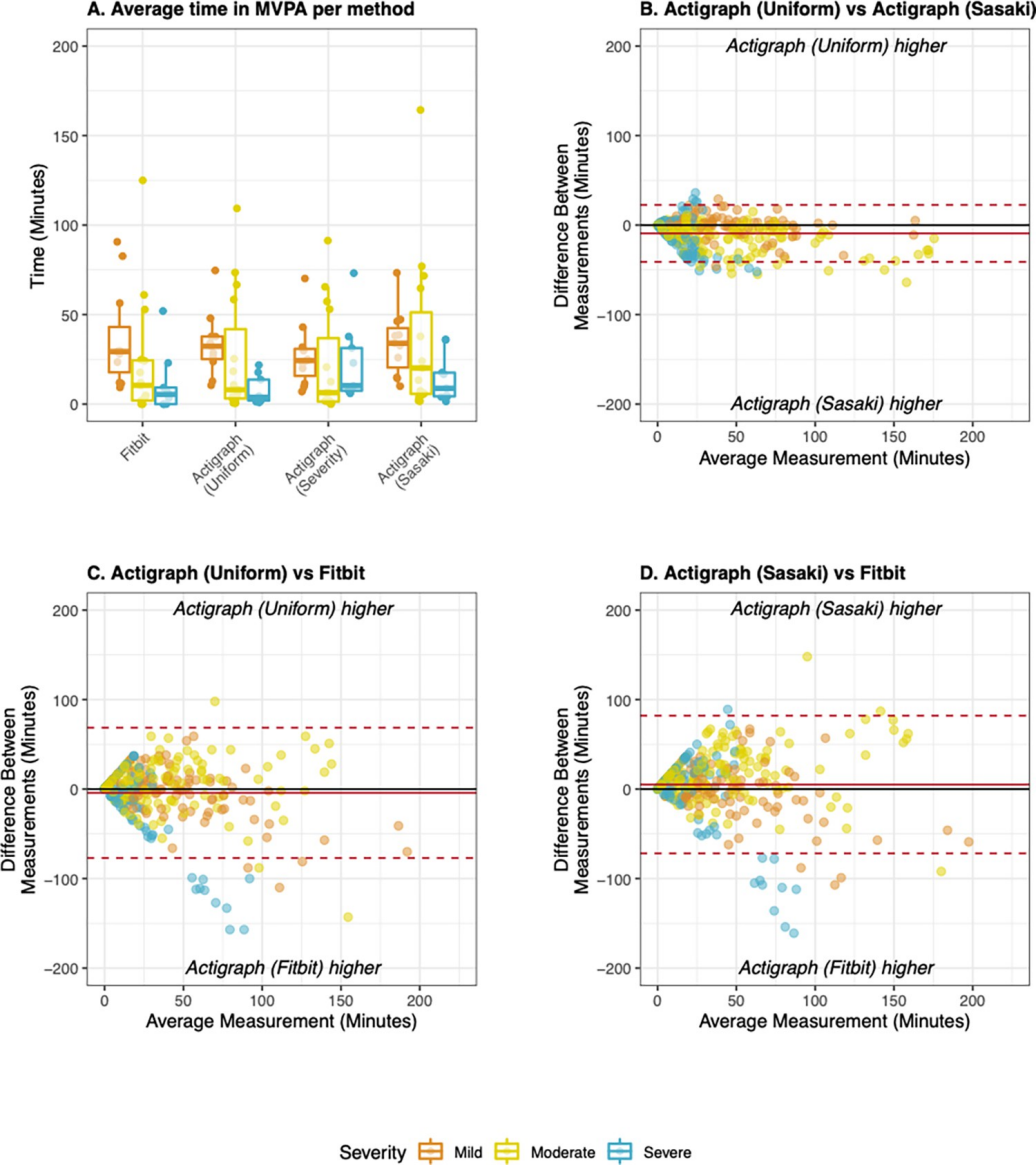
Time in moderate to vigorous physical activity derived from Fitbit and Actigraph during free living evaluation. A) Average time in PA derived from the Fitbit, Actigraph (Uniform), and Actigraph (Sasaki) stratified by disease severity. B) Bland Altman plot comparing both Actigraph methods to each other; C) Bland Altman plot comparing Fitbit to Actigraph (Uniform), D) Bland Altman plot comparing Fitbit to Actigraph (Sasaki). All Bland Altman plots display data collected during rehab and at home at the daily level of aggregation. Mean bias (solid line) and limits of agreement (dotted lines) were adjusted for patient-level random effects. Mild MS: EDSS < 4.0, Moderate MS: EDSS 4.0–5.5, Severe MS: EDSS 6.0–6.5.

During free-living evaluations, overall pairwise agreement between Fitbit and Actigraph methods was poor to fair at the epoch (k: 0.39–0.42) level, fair at the daily level (CCC: 0.41–0.45), and good to excellent at the average level (CCC: 0.63–0.80) (Table 4). Bland Altman analysis showed that median bias between methods was low in all cases, though limits of agreement between methods were wider when Fitbit was compared to Actigraph methods than when Actigraph methods were compared to each other (Fig 7). Agreement differed across disease severity strata at all levels of aggregation. Agreement was consistently highest in those with moderate MS, slightly lower in those with mild MS, and lowest in those with severe MS (S4 Table).

#### 3. Time in MVPA: Did Fitbit correlate with criterion measures?

During free-living evaluation, correlations between Fitbit and Actigraph methods were moderate at the daily level (r: 0.65–0.68) and moderate to strong at the average level (r: 0.64–0.80) (Table 4). Correlations were highest in the subgroup with moderate MS, lower in the subgroup with mild MS, and lowest in the subgroup with severe MS (S4 Table).

#### 4. Time in MVPA: Did Fitbit associate with clinical outcomes?

Fitbit-derived time in MVPA exhibited moderate to strong correlations with clinical outcome measures, whereas the Actigraph methods often did not (Table 3). Fitbit exhibited the expected differences between groups based on MSWS-12 cutoffs, but differences between mild/ moderate and severe MS did not reach statistical significance. All Actigraph-derived PA estimates exhibited differences between subgroups (Table 3).

### Triangulating the validity of Fitbit-derived PA metrics

Qualitative ratings generated through the triangulation process are shown in Table 5. Fitbit and Actigraph-derived PA metrics cannot be considered equivalent. Nor can most Actigraph methods be considered equivalent to each other. However, all measures exhibited evidence of construct validity.

**Table 5:** Triangulating the validity of Fitbit-derived PA metrics.

	Step count	Time in PA	Time in MVPA
***Did criterion measures agree with each other*?**			
Scripted tasks	+	++	++
Free living, Epoch level	--	++	+/++
Free living, Daily level	--	+	+
Free living, Average level	-	-	++
Consistency across severity strata*	+	+	-
***Did criterion measures correlate with each other*?**			
Scripted tasks	++	na	na
Free living, Epoch level	-	na	na
Free living, Daily level	++	++	++
Free living, Average level	++	++	++
Consistency across severity strata*	+	+	-
***Did Fitbit agree with criterion measures*?**			
Scripted tasks	+	++	--
Free living, Epoch level	--	+/++	-/+
Free living, Daily level	-/+	--/-	+
Free living, Average level	+	-/+	+/++
Consistency across severity strata*	-	-	-
Did Fitbit meet or exceed the agreement exhibited by criterion measures?	+	-/+	-
***Did Fitbit correlate with criterion measures*?**			
Scripted tasks	++	na	na
Free living, Epoch level	-	na	na
Free living, Daily level	++	+/++	+
Free living, Average level	++	+/++	+/++
Consistency across severity strata*	-/+	-	-
Did Fitbit meet or exceed the correlations exhibited by criterion measures?	+	-/+	-
***Did Fitbit relate to clinical outcomes*?**			
Did Fitbit exhibit the expected correlations with clinical measures?	+	+	+
Did Fitbit-derived PA metrics differ across known groups?	+	-	+
Did Fitbit meet or exceed the relationships exhibited by criterion measures?	+	+	+
***Validity of Fitbit-derived PA metrics***			
Can criterion measures be considered equivalent?	-	-	-
Can Fitbit and Actigraph be considered equivalent?	-	-	-
Did Fitbit exhibit evidence of construct validity?	+	+	+

++: Excellent agreement or strong correlations (0.75–1.0)

+: Fair to good agreement or moderate correlations (0.4–0.75)

-: Poor agreement, weak correlations (0.2–0.4)

—: Very weak or complete lack of agreement or correlation (<0.2)

+/-: Evidence was mixed; Binary yes/no responses are indicated with + or -, respectively

*Consistency across severity strata describes whether the trends observed during scripted tasks and at the epoch, daily, and average levels were consistent across subgroups with mild, moderate, and severe MS. A positive rating in this category does not necessarily mean that measures correlated or agreed with each other.

## Discussion

In this study, we evaluated the validity of PA metrics derived from the Fitbit Inspire HR during scripted walking tasks and free-living activity at multiple levels of data aggregation. Fitbit-derived PA metrics demonstrated construct validity, but not equivalency with criterion measures derived from the Actigraph GT3X. Correlations and agreement between measures differed across settings, data aggregation levels, and disease severity strata. However, criterion measures exhibited limited agreement amongst themselves, and we demonstrate that, in most cases, Fitbit performs within the range of their inter-method variability. In light of these findings, consumer-grade fitness trackers such as Fitbit may be advantageous for long-term PA tracking in PwMS.

### Evaluating the validity of Fitbit-derived physical activity metrics

#### Step count

Our triangulation suggests that Fitbit-derived step count may outperform Actigraph-derived step count during free-living PA in people with mild or moderate MS, but should be used with caution in those with severe walking impairment. We found that Fitbit-derived step counts exhibited strong correlations but poor agreement with Actigraph-derived step counts. This is consistent with previous studies comparing Fitbit-derived step counts to those derived from Actigraph in healthy populations [77–80] and MS populations [30,31]. However, Actigraph-derived step counts often exhibited worse agreement with each other than they did with the Fitbit, and step counts derived from the Actigraph (LFE) were considered unrealistically high for this population by clinical experts (authors JK, RG). This pattern is consistent with a previous investigation, in which Actigraph (Standard) underestimated step count by 25–30%, and Actigraph (LFE) overestimated step count by 30% [33]. Therefore the ‘true’ step count likely falls somewhere between these two metrics, as Fitbit-derived step counts did in this study. Fitbit demonstrated different sources of error than Actigraph methods during scripted tasks which disproportionately impacted persons with severe MS. This reduced performance has been previously attributed to reductions in walking speed [59]. However, our observations suggest that wheelchair or rollator use and common balance management strategies such as holding on to furniture for support [81,82] affect upper body movement while walking and may contribute to reduced performance in those with severe MS.

#### Time in physical activity

Fitbit-derived time in PA was consistently lower than both Actigraph methods, though the three correlated strongly and exhibited moderate to strong agreement at most levels of data aggregation. Neither Actigraph method has been validated under free-living conditions. Therefore, it is unclear how they relate to “true” time in free-living physical activity, defined as “any voluntary bodily movement produced by the skeletal muscles that requires energy expenditure” by the World Health Organization [83]. We offer three potential explanations for the differences between Actigraph and Fitbit-derived time in PA. First, Fitbit’s sensitivity to PA may simply be reduced in populations with MS, as PwMS have altered gait compared to healthy controls [26,27]. Alternatively, Fitbit’s mischaracterization of activity related to upper rather than lower body motion may introduce different biases into Fitbit and Actigraph-derived measurements, as it did for step count. Finally, the inclusion of heart rate in Fitbit’s PA algorithms may yield measurements of a highly related, but slightly different PA construct than that measured by Actigraph. Nevertheless, Fitbit exhibited greater evidence of construct validity in this study (Table 3). The criterion validity of all three methods should be confirmed in future work.

#### Time in moderate to vigorous physical activity

Fitbit-derived MVPA exhibited no agreement with Actigraph-derived MVPA during scripted tasks and only poor to fair agreement at the epoch or daily level. Previous studies in healthy populations similarly suggest disagreement between the Fitbit the Actigraph. Two recent systematic reviews of Fitbit validation studies suggest that, in healthy populations, Fitbit strongly correlates with Actigraph-derived MVPA [19], though point estimates of MVPA derived from the Fitbit overestimate time in MVPA compared to the Actigraph [20]. On the surface, our findings align with these findings. However, in this study, Fitbit-derived MVPA exhibited evidence of convergent and known-groups validity, whereas Actigraph-derived PA metrics often did not. This, and the fact that Fitbit did not register scripted activities as MVPA, suggest that Fitbit-derived MVPA reflects a different construct than Actigraph-derived MVPA. The differences between these constructs could not be characterized based on the evidence generated in this study, but may relate to the inclusion of heart rate and upper body PA in Fitbit’s activity intensity assessment algorithms.

### The case for consumer-grade activity monitors

If Fitbit-derived metrics do indeed exhibit an acceptable level of construct validity in PwMS, they present new opportunities as long-term, engaging, and user-friendly PA monitoring tools. Current monitoring practices rely on questionnaires, diaries, or research-grade wearable devices. However, the user experiences and validity of these methods are also limited. Questionnaires and diaries are burdensome to complete regularly, subject to recall bias, and insensitive to short bouts of light or lifestyle physical activity [84–87]. Participants in previous studies have reported that research-grade wearable devices are “bulky,” “uncomfortable,” and “attract unwanted attention” during free-living PA tracking [88]. Further, PwMS prefer to receive feedback about their activity from devices that they wear during studies [89,90], which the Actigraph and many other research-grade accelerometers do not provide [88]. Conversely, Fitbit devices are considered comfortable and inconspicuous [91]. They collect data passively and provide regular feedback to the wearer, potentially increasing long-term engagement with PA monitoring [17,92]. We provisionally demonstrated this effect in this study, as Fitbit wear time was higher than that of the Actigraph. Finally, the Fitbit Inspire HR is relatively affordable compared to research grade devices at a price of approximately 100 US dollars [93]. Fitbit-derived PA metrics may not be fit for all research purposes, for example as outcome measures in efficacy studies which may be confounded by the device’s feedback. Nevertheless, the rich, longitudinal data derived from Fitbit devices could reveal novel insights and patterns not discoverable through current PA assessments.

### Strengths, limitations, and future work

This study investigated the validity of Fitbit-derived physical activity metrics according to best practices, accounting for known shortcomings of widely-used reference measurements. It explored the construct validity of three Fitbit-derived PA metrics in a systematic manner, in multiple settings, patient subgroups, and levels of data aggregation. It therefore represents, to the authors’ knowledge, the most comprehensive evaluation of a Fitbit’s validity to date.

However, this study is not without its limitations. Johnston et al. recommend video monitoring as a criterion measure for step counts during free-living evaluation, though they note that this method is frequently infeasible due to processing time and patient burden [32]. We opted not to use video during free-living evaluation, instead addressing the known shortcomings of available criterion methods through triangulation [57]. Similarly, we did not use calorimetry to derive PA intensity during any tasks, as these are difficult and burdensome to implement as criterion measures in contexts other than scripted walking. We therefore cannot quantify the Fitbit’s absolute accuracy through the present study, and our findings should be considered relative to the known benefits and shortcomings of Actigraph methods.

The findings presented here are necessary, but not sufficient, to support the use of Fitbit-derived PA metrics for MS. If Fitbit-derived metrics are to be used to self-manage PA, track PA over time, or evaluate the efficacy of novel interventions, they must be able to detect change at the patient and population level. It is possible that the biases demonstrated here could impact Fitbit-derived metrics’ ability to detect change on both the individual and population level, and future work should evaluate their responsiveness. Novel analysis methods which can account for these confounding effects, especially those which capitalize on the richness of long-term PA data, should also be the subject of future research.

## Conclusions

Fitbit-derived metrics are not equivalent to those derived from Actigraph. However, they exhibit similar or stronger evidence of construct validity. Consumer-grade fitness trackers such as the Fitbit may therefore be suitable as PA management tools for people with mild or moderate MS–particularly to monitor intra-individual temporal changes. However, they should be used with caution in populations with advanced walking impairment. Future work should investigate the criterion validity and responsiveness of both Fitbit and Actigraph-derived PA metrics.

## Supporting information

S1 Text Standard operating procedure: Manual step counts. (DOCX)

S1 Fig Epoch-level agreement between Fitbit-derived and Actigraph-derived step count. A) between Fitbit and Actigraph (Standard), B) between Fitbit and Actigraph (LFE), and C) between both Actigraph methods. Each point represents the number of steps counted in a 5-minute epoch. Epochs in perfect agreement fall along the black diagonal line. The majority of epochs were relatively consistent, though not in perfect agreement, and fell near the diagonal. A relatively small portion of epochs exhibited very low counts by one device and high counts by the other device. Two percent (Standard filter, panel A) and four percent (LFE, panel B) of exhibited this distribution. This discrepancy may be related to the limited specificity of the wear time algorithm, the limited ability of the Actigraph (Standard) method to detect impaired gait, or the differing sources of bias between devices the devices. In panel C, all points are above the diagonal because the LFE always yielded step counts which were equal to or greater than those with the standard filter.
(DOCX)

S1 Table Participant characteristics during each stage of the validity evaluation. (DOCX)

S2 Table Correlation and agreement between step counts derived from Fitbit and Actigraph. (DOCX)

S3 Table Correlation and agreement between time in physical activity derived from Fitbit and Actigraph. (DOCX)

S4 Table Correlation and agreement between time in moderate to vigorous physical activity derived from Fitbit and Actigraph. (DOCX)
