# Supplementary material for: Non-equivalent, but still valid: Establishing the construct validity of a consumer fitness tracker in persons with multiple sclerosis
Source: PLOS Digit Health. 2023 Jan 25;2(1):e0000171. doi: 10.1371/journal.pdig.0000171 (PMC9931345; doi:10.1371/journal.pdig.0000171)
Supplement: S1 Text — (DOCX) [file pdig.0000171.s001.docx]

**S1 Text,**

**Standard operating procedure: Manual step counts**

Average manual step counts were considered the criterion measure for assessing the validity of Fitbit’s step count algorithm during scripted tests. Laboratory and semi-free-living evaluations were video-recorded and two assessors manually counted steps according to the following standard operating procedure:

**What is a step?**

- Human ***walking*** is a method of locomotion and is defined as initiating and maintaining a forward displacement of the centre of mass in an intended direction involving the use of the two legs which provide both support and propulsion. The feet are repetitively and reciprocally lifted and set down whereby at least one foot is in contact with the ground at all times.^9,10^ ***Walking*** with walking aids is included in this definition.
- A ***step*** is the interval between the initial contacts of the ipsi- and contralateral foot.^9^
- We count the following as steps:
  - Stepping in place
  - Steps taken to regain balance
  - The foot must leave the ground completely to be counted as a step
- We do not count the following as steps:
  - Returning feet to the ground during/after a postural transition
  - Non-weight-bearing relocations of the feet (for example, while sitting)

**Counting Steps**

- All counters should undergo the consistency check described below.
- Watch the video of each scripted task at normal speed, or slower than normal speed if needed.
- Use a counting tool (such as hand-held counter or an online tool)
- Every step observed in the video should be counted, even during rest periods.  Count steps separately for each task and rest period. Log steps as described by the forms below. Record notes and observations as needed.
- Two counters count steps independently for each video.

**Consistency Check**

- Counters will independently count steps in one video of each scripted task.
- Steps and situations which are unclear will be clarified through discussion, according to the “eligibility” criteria for steps above.
- This process will be repeated on new videos until counts differ by 5% or less for each task

| Manual Step count report form: Scripted, Semi-free-living tasks | |
| --- | --- |
| Participant ID |  |
| Start time (Rest 1) |  |
| End time (Rest 6) |  |
|  | **Steps** |
| Rest 1 |  |
| Walk with postural transitions |  |
| Rest 2 |  |
| Simulated cleaning |  |
| Rest 3 |  |
| Sit to stand |  |
| Rest 4 |  |
| Wheelchair push |  |
| Rest 5 |  |
| Stair climbing |  |
| Rest 6 |  |
| Notes |  |

| Manual Step count report form: Six minute walk test | |
| --- | --- |
| Participant ID |  |
| Start time (Rest 1) |  |
| End time (Rest 2) |  |
| 6MWT (Steps) |  |
| Notes |  |
