# Supplementary material for: Non-equivalent, but still valid: Establishing the construct validity of a consumer fitness tracker in persons with multiple sclerosis
Source: PLOS Digit Health. 2023 Jan 25;2(1):e0000171. doi: 10.1371/journal.pdig.0000171 (PMC9931345; doi:10.1371/journal.pdig.0000171)
Supplement: S1 Fig — A) between Fitbit and Actigraph (Standard), B) between Fitbit and Actigraph (LFE), and C) between both Actigraph methods. Each point represents the number of steps counted in a 5-minute epoch. Epochs in perfect agreement fall along the black diagonal line. The majority of epochs were relatively consistent, though not in perfect agreement, and fell near the diagonal. A relatively small portion of epochs exhibited very low counts by one device and high counts by the other device. Two percent (Standard filter, panel A) and four percent (LFE, panel B) of exhibited this distribution. This discrepancy may be related to the limited specificity of the wear time algorithm, the limited ability of the Actigraph (Standard) method to detect impaired gait, or the differing sources of bias between devices the devices. In panel C, all points are above the diagonal because the LFE always yielded step counts which were equal to or greater than those with the standard filter. (DOCX) [file pdig.0000171.s002.docx]

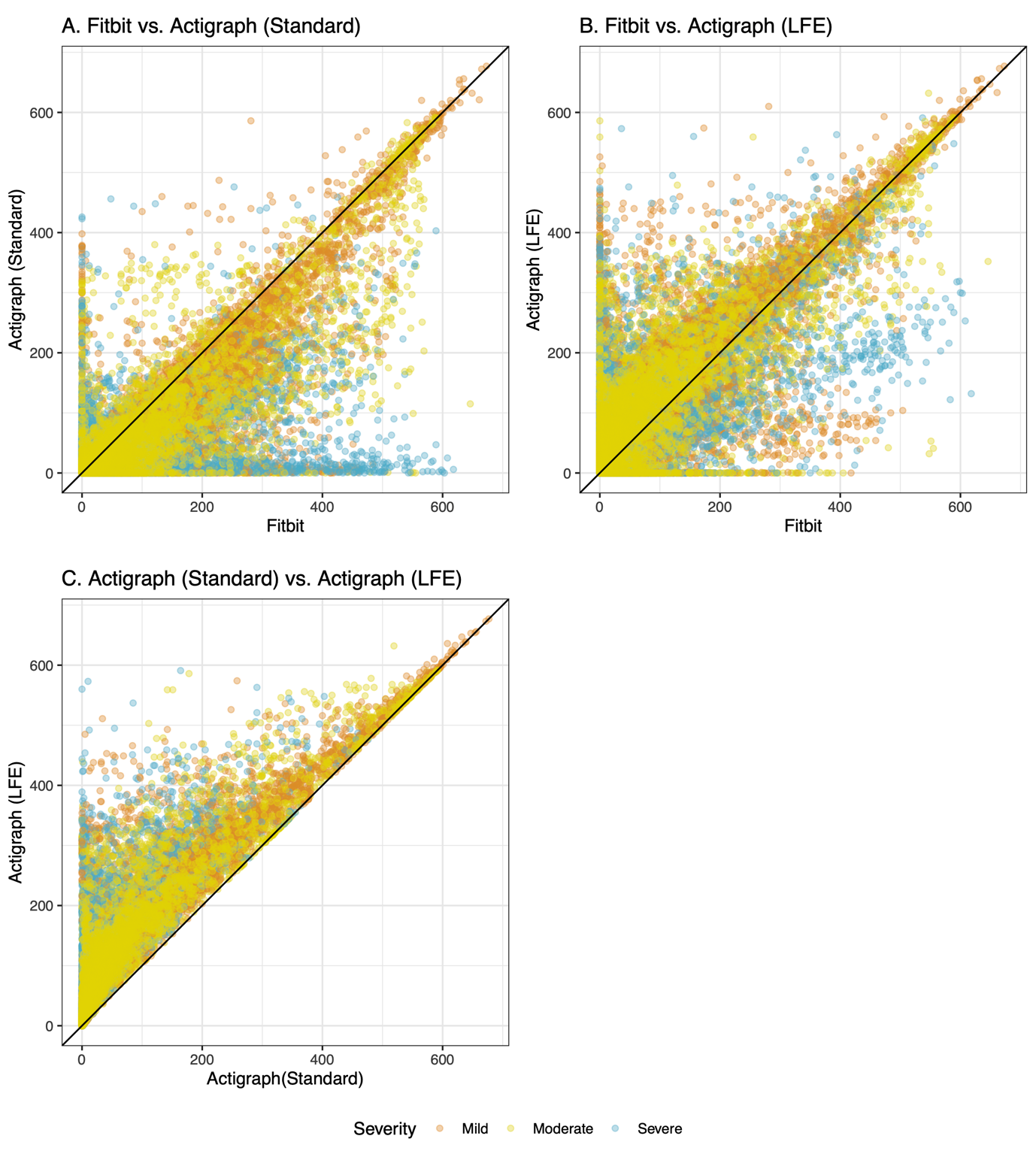


**S1 Fig:** **Epoch-level agreement between Fitbit-derived and Actigraph-derived step count.** A) between Fitbit and Actigraph (Standard), B) between Fitbit and Actigraph (LFE), and C) between both Actigraph methods. Each point represents the number of steps counted in a 5-minute epoch. Epochs in perfect agreement fall along the black diagonal line. The majority of epochs were relatively consistent, though not in perfect agreement, and fell near the diagonal. A relatively small portion of epochs exhibited very low counts by one device and high counts by the other device. Two percent (Standard filter, panel A) and four percent (LFE, panel B) of exhibited this distribution. This discrepancy may be related to the limited specificity of the wear time algorithm, the limited ability of the Actigraph (Standard) method to detect impaired gait, or the differing sources of bias between devices the devices. In panel C, all points are above the diagonal because the LFE always yielded step counts which were equal to or greater than those with the standard filter.
