# Supplementary material for: Non-equivalent, but still valid: Establishing the construct validity of a consumer fitness tracker in persons with multiple sclerosis
Source: PLOS Digit Health. 2023 Jan 25;2(1):e0000171. doi: 10.1371/journal.pdig.0000171 (PMC9931345; doi:10.1371/journal.pdig.0000171)
Supplement: S1 Table — (DOCX) [file pdig.0000171.s003.docx]

| S1 Table: **Participant characteristics during each stage of the validity evaluation.** | | | | |
| --- | --- | --- | --- | --- |
| **Comparison** | **Laboratory evaluation** | **Semi-free-living evaluation** | **Free living evaluation**  **(Epoch & daily level aggregation)** | **Free living evaluation (Average level aggregation)*** |
| N | 35 | 12 | 42 | 35 |
| Age | 45 [40-51] | 48 [45-51] | 46 [40-51] | 44 [40-50] |
| Sex N Females, (%) | 25 (71.4) | 10 (83.3) | 27 (64.3) | 25 (71.4) |
| EDSS | 4 [3.5-5.75] | 4.5 [3.375-6] | 4.5 [3.5-6] | 4 [3.5-5.5] |
| Mild | 12 | 4 | 13 | 13 |
| Moderate | 14 | 4 | 17 | 14 |
| Severe | 9 | 4 | 12 | 8 |
| MSWS-12 | 40.6 [25.0-59.9] | 46.9 [23.4-74.0] | 52.1 [27.1-66.7] | 40.6 [25.5-63.5] |
| 6MWT | 405 [271.5-430] | 430 [226-474.5] | 345 [242-428] | 370 [275-430] |
| No walking aid | 19 | 5 | 22 | 19 |
| 2 Sticks | 6 | 1 | 9 | 6 |
| Rollator | 1 | 0 | 1 | 1 |
| Other | 1 | 1 | 1 | 1 |
| 10mGS | 9 [7-11] | 11 [7-15] | 9 [7-13] | 9 [7-11] |
| No walking aid | 22 | 6 | 26 | 23 |
| 2 Sticks | 5 | 2 | 8 | 5 |
| Rollator | 2 | 1 | 2 | 2 |
| Other | 4 | 2 | 4 | 3 |
| Values are shown as N or median [IQR] unless otherwise specified  * Differs from Epoch and Daily-level aggregation because 7 additional participants did not meet minimum wear time requirements in the home setting, though they did meet minimum wear time requirements overall. | | | | |
