# Supplementary material for: Non-equivalent, but still valid: Establishing the construct validity of a consumer fitness tracker in persons with multiple sclerosis
Source: PLOS Digit Health. 2023 Jan 25;2(1):e0000171. doi: 10.1371/journal.pdig.0000171 (PMC9931345; doi:10.1371/journal.pdig.0000171)
Supplement: S2 Table — (DOCX) [file pdig.0000171.s004.docx]

| **S2 Table. Correlation and agreement between step counts derived from Fitbit and Actigraph.** | | | | | | | | |
| --- | --- | --- | --- | --- | --- | --- | --- | --- |
|  | **Overall** | | **Mild** | | **Moderate** | | **Severe** | |
| **Comparison** | **r [95% CI]** | **CCC [95% CI]** | **r [95% CI]** | **CCC [95% CI]** | **r [95% CI]** | **CCC [95% CI]** | **r [95% CI]** | **CCC [95% CI]** |
| ***Scripted tasks*** | | | | | | | | |
| Manual vs Act(Stand)^a^ | 0.97 [0.77 - 0.99] | 0.68 [0.37 - 0.82] |  |  |  |  |  |  |
| Manual vs Act(LFE)^a^ | 0.98 [0.78 - 0.99] | 0.73 [0.13 - 0.84] |  |  |  |  |  |  |
| Act(Stand) vs Act(LFE)^a^ | 0.97 [0.85 - 0.99] | 0.60 [0.06 - 0.76] |  |  |  |  |  |  |
| Fitbit vs. Manual | 0.92 [0.63 - 0.98] | 0.66 [0.14 - 0.80] |  |  |  |  |  |  |
| Fitbit vs. Act(Stand) | 0.92 [0.65 - 0.97] | 0.55 [0.08 - 0.72] |  |  |  |  |  |  |
| Fitbit vs. Act(LFE) | 0.93 [0.65 - 0.98] | 0.65 [0.24 - 0.77] |  |  |  |  |  |  |
| ***Free living, Epoch level*** | | | | | | | | |
| Act(Stand) vs Act(LFE) ^a^ | 0.27 [0.24 - 0.30] | 0.03 [0.02 - 0.04] | 0.20 [0.17 - 0.24] | *0.01 [0.00 - 0.02]* | 0.26 [0.23 - 0.30] | 0.03 [0.02 - 0.05] | 0.35 [0.32 - 0.38] | 0.03 [0.01 - 0.04] |
| Fitbit vs. Act(Stand) | 0.22 [0.19 - 0.25] | 0.04 [0.03 - 0.05] | 0.15 [0.08 - 0.21] | *0.02 [0.00 - 0.04]* | 0.22 [0.18 - 0.26] | 0.04 [0.02 - 0.06] | 0.25 [0.15 - 0.32] | 0.04 [0.02 - 0.08] |
| Fitbit vs. Act(LFE) | 0.22 [0.19 - 0.25] | 0.03 [0.02 - 0.05] | 0.18 [0.13 - 0.22] | *0.02 [0.00 - 0.04]* | 0.21 [0.17 - 0.25] | 0.03 [0.02 - 0.05] | 0.25 [0.19 - 0.32] | 0.04 [0.01 - 0.07] |
| ***Free living, Daily level*** | | | | | | | | |
| Act(Stand) vs Act(LFE) ^a^ | 0.88 [0.84 - 0.91] | 0.15 [0.11 - 0.20] | 0.82 [0.78 - 0.86] | 0.08 [0.02 - 0.14] | 0.87 [0.79 - 0.93] | 0.18 [0.10 - 0.27] | 0.92 [0.91 - 0.95] | 0.08 [0.04 - 0.12] |
| Fitbit vs. Act(LFE) | 0.82 [0.78 - 0.87] | 0.33 [0.22 - 0.43] | 0.78 [0.69 - 0.84] | 0.24 [0.04 - 0.41] | 0.82 [0.72 - 0.90] | 0.34 [0.21 - 0.48] | 0.82 [0.70 - 0.93] | 0.28 [0.05 - 0.57] |
| Fitbit vs. Act(Stand) | 0.80 [0.75 - 0.85] | 0.44 [0.32 - 0.57] | 0.71 [0.50 - 0.79] | 0.34 [0.15 - 0.48] | 0.82 [0.71 - 0.90] | 0.48 [0.29 - 0.63] | 0.73 [0.47 - 0.84] | 0.28 [0.13 - 0.50] |
| ***Free living, Average level*** | | | | | | | | |
| Act(Stand) vs Act(LFE) ^a^ | 0.89 [0.78 - 0.94] | 0.27 [0.16 - 0.37] | 0.81 [0.46 - 0.94] | 0.13 [0.02 - 0.25] | 0.92 [0.76 - 0.97] | 0.33 [0.13 - 0.51] | 0.86 [0.39 - 0.97] | *0.14 [-0.01 - 0.28]* |
| Fitbit vs. Act(LFE) | 0.86 [0.74 - 0.93] | 0.50 [0.34 - 0.63] | 0.89 [0.66 - 0.97] | 0.38 [0.14 - 0.57] | 0.94 [0.81 - 0.98] | 0.60 [0.35 - 0.78] | *0.65 [-0.11 - 0.93]* | *0.32 [-0.08 - 0.63]* |
| Fitbit vs. Act(Stand) | 0.82 [0.67 - 0.90] | 0.65 [0.47 - 0.77] | 0.88 [0.63 - 0.96] | 0.58 [0.30 - 0.76] | 0.94 [0.81 - 0.98] | 0.75 [0.52 - 0.88] | *0.35 [-0.47 - 0.85]* | *0.19 [-0.23 - 0.55]* |
| ^a^ Comparison between two criterion measures  Point estimates which did not reach statistical significance, defined here as the 95% confidence intervals excluding 0, are shown in grey italics.  *Act: Actigraph; Stand: Standard; LFE: Low frequency extension; r: Pearson correlation coefficient; CI: confidence interval; CCC: Lin’s Concordance correlation coefficient* | | | | | | | | |
