# Supplementary material for: Non-equivalent, but still valid: Establishing the construct validity of a consumer fitness tracker in persons with multiple sclerosis
Source: PLOS Digit Health. 2023 Jan 25;2(1):e0000171. doi: 10.1371/journal.pdig.0000171 (PMC9931345; doi:10.1371/journal.pdig.0000171)
Supplement: S3 Table — (DOCX) [file pdig.0000171.s005.docx]

| **S3 Table: Correlation and agreement between time in physical activity derived from Fitbit and Actigraph.** | | | | | | |
| --- | --- | --- | --- | --- | --- | --- |
|  | **Scripted tasks** | **Free living,**  **Epoch level** | **Free living,**  **Daily level** | | **Free living,**  **Average level** | |
| **Comparison** | **k [95% CI]** | **k [95% CI]** | **r [95% CI]** | **CCC [95% CI]** | **r [95% CI]** | **CCC [95% CI]** |
| ***Overall*** | | | | | | |
| Act(VM) vs Act(Vert) ^a^ | 0.93 [0.88 - 0.97] | 0.75 [0.73 - 0.77] | 0.78 [0.74 - 0.83] | 0.34 [0.25 - 0.41] | 0.92 [0.85 - 0.96] | 0.71 [0.57 - 0.81] |
| Act(Vert) vs Fitbit | 0.87 [0.72 - 1.02] | 0.76 [0.73 - 0.80] | 0.74 [0.67 - 0.80] | 0.36 [0.21 - 0.50] | 0.72 [0.50 - 0.85] | 0.52 [0.32 - 0.68] |
| Act(VM) vs Fitbit | 0.85 [0.71 - 0.99] | 0.62 [0.58 - 0.66] | 0.82 [0.77 - 0.86] | 0.18 [0.11 - 0.26] | 0.76 [0.57 - 0.87] | 0.35 [0.20 - 0.49] |
| ***Mild*** | | | | | | |
| Act(VM) vs Act(Vert) ^a^ | 0.96 [0.92 - 1.00] | 0.75 [0.71 - 0.80] | 0.75 [0.63 - 0.83] | 0.31 [0.18 - 0.45] | 0.92 [0.75 - 0.98] | 0.62 [0.33 - 0.80] |
| Act(Vert) vs Fitbit | 0.89 [0.72 - 1.05] | 0.80 [0.78 - 0.82] | 0.77 [0.62 - 0.85] | 0.43 [0.13 - 0.64] | 0.89 [0.66 - 0.97] | 0.61 [0.32 - 0.80] |
| Act(VM) vs Fitbit | 0.85 [0.69 - 1.00] | 0.66 [0.62 - 0.70] | 0.83 [0.74 - 0.89] | 0.25 [0.08 - 0.39] | 0.87 [0.62 - 0.96] | 0.39 [0.14 - 0.60] |
| ***Moderate*** | | | | | | |
| Act(VM) vs Act(Vert) ^a^ | 0.90 [0.78 - 1.01] | 0.78 [0.74 - 0.81] | 0.78 [0.68 - 0.86] | 0.35 [0.16 - 0.50] | 0.94 [0.81 - 0.98] | 0.76 [0.52 - 0.89] |
| Act(Vert) vs Fitbit | 0.91 [0.77 - 1.04] | 0.80 [0.79 - 0.82] | 0.72 [0.57 - 0.82] | 0.38 [0.22 - 0.51] | 0.83 [0.54 - 0.95] | 0.69 [0.37 - 0.86] |
| Act(VM) vs Fitbit | 0.92 [0.85 - 0.99] | 0.67 [0.64 - 0.71] | 0.79 [0.68 - 0.87] | 0.18 [0.10 - 0.28] | 0.87 [0.63 - 0.96] | 0.46 [0.19 - 0.66] |
| ***Severe*** | | | | | | |
| Act(VM) vs Act(Vert) ^a^ | 0.93 [0.88 - 0.98] | 0.71 [0.66 - 0.75] | 0.81 [0.65 - 0.89] | 0.32 [0.13 - 0.50] | 0.89 [0.49 - 0.98] | 0.68 [ 0.24 - 0.89] |
| Act(Vert) vs Fitbit | 0.80 [0.57 - 1.02] | 0.64 [0.54 - 0.74] | 0.70 [0.62 - 0.84] | 0.17 [0.04 - 0.50] | *0.38 [-0.44 - 0.86]* | -- |
| Act(VM) vs Fitbit | 0.75 [0.54 - 0.96] | 0.49 [0.36 - 0.61] | 0.84 [0.77 - 0.92] | 0.08 [0.02 - 0.24] | *0.48 [-0.33 - 0.89]* | *0.14 [-0.10 - 0.36]* |
| ^a^ Comparison between two criterion measures  Point estimates which did not reach statistical significance, defined here as the 95% confidence intervals excluding 0, are shown in grey italics. For one analysis, ties and a small sample size prevented the CCC from being calculated (shown as --)  *Act: Actigraph; Vert: Vertical; VM: Vector Magnitude; k: Fleiss’ kappa; CI: confidence interval; r: Pearson correlation coefficient; CCC: Lin’s Concordance correlation coefficient* | | | | | | |
