# Supplementary material for: Non-equivalent, but still valid: Establishing the construct validity of a consumer fitness tracker in persons with multiple sclerosis
Source: PLOS Digit Health. 2023 Jan 25;2(1):e0000171. doi: 10.1371/journal.pdig.0000171 (PMC9931345; doi:10.1371/journal.pdig.0000171)
Supplement: S4 Table — (DOCX) [file pdig.0000171.s006.docx]

| **S4 Table: Correlation and agreement between time in moderate to vigorous physical activity derived from Fitbit and Actigraph.** | | | | | | |
| --- | --- | --- | --- | --- | --- | --- |
|  | **Scripted tasks** | **Free living,**  **Epoch level** | **Free living,**  **Daily level** | | **Free living,**  **Average level** | |
| **Comparison** | **k [95% CI]** | **k [95% CI]** | **r [95% CI]** | **CCC [95% CI]** | **r [95% CI]** | **CCC [95% CI]** |
| ***Overall*** | | | | | | |
| Act(Uni) vs Act(Sev) ^a^ | 0.84 [0.77 - 0.91] | 0.82 [0.75 - 0.89] | 0.79 [0.68 - 0.86] | 0.63 [0.46 - 0.74] | 0.89 [0.79 - 0.94] | 0.87 [ 0.77 - 0.93] |
| Act(Uni) vs Act(Sasaki) ^a^ | 0.79 [0.63 - 0.94] | 0.73 [0.66 - 0.80] | 0.82 [0.72 - 0.88] | 0.63 [0.47 - 0.73] | 0.90 [0.82 - 0.95] | 0.88 [ 0.78 - 0.94] |
| Act(Sev) vs Act(Sasaki) ^a^ | 0.76 [0.63 - 0.88] | 0.68 [0.60 - 0.76] | 0.78 [0.65 - 0.85] | 0.56 [0.38 - 0.66] | 0.76 [0.57 - 0.87] | 0.72 [ 0.53 - 0.84] |
| Act(Uni) vs Fitbit | *-0.18 [-0.48 - 0.13]* | 0.39 [0.27 - 0.51] | 0.68 [0.56 - 0.80] | 0.45 [0.27 - 0.63] | 0.80 [0.64 - 0.90] | 0.80 [ 0.64 - 0.89] |
| Act(Sev) vs Fitbit | *-0.18 [-0.48 - 0.13]* | 0.42 [0.31 - 0.54] | 0.65 [0.55 - 0.75] | 0.41 [0.27 - 0.56] | 0.64 [0.40 - 0.81] | 0.63 [ 0.39 - 0.79] |
| Act(Sasaki) vs Fitbit | *-0.18 [-0.46 - 0.10]* | 0.41 [0.31 - 0.52] | 0.67 [0.54 - 0.79] | 0.44 [0.27 - 0.58] | 0.79 [0.62 - 0.89] | 0.78 [ 0.61 - 0.88] |
| ***Mild*** | | | | | | |
| Act(Uni) vs Act(Sev) ^a^ | 0.80 [0.66 - 0.94] | 0.90 [0.87 - 0.92] | 0.66 [0.55 - 0.78] | 0.40 [0.27 - 0.56] | 0.99 [0.97 - 1.00] | 0.89 [ 0.68 - 0.96] |
| Act(Uni) vs Act(Sasaki) ^a^ | 0.87 [0.77 - 0.97] | 0.81 [0.75 - 0.87] | 0.64 [0.55 - 0.75] | 0.41 [0.30 - 0.56] | 0.89 [0.67 - 0.97] | 0.93 [ 0.83 - 0.97] |
| Act(Sev) vs Act(Sasaki) ^a^ | 0.86 [0.73 - 0.98] | 0.79 [0.72 - 0.87] | 0.65 [0.56 - 0.77] | 0.37 [0.25 - 0.52] | 0.85 [0.57 - 0.95] | 0.78 [ 0.47 - 0.92] |
| Act(Uni) vs Fitbit | *-0.22 [-0.69 - 0.25]* | 0.45 [0.33 - 0.57] | 0.58 [0.37 - 0.75] | 0.34 [0.13 - 0.56] | 0.57 [0.03 - 0.85] | 0.53 [ 0.06 - 0.80] |
| Act(Sev) vs Fitbit | *-0.23 [-0.70 - 0.24]* | 0.49 [0.36 - 0.61] | 0.59 [0.37 - 0.75] | 0.32 [0.12 - 0.55] | 0.54 [-0.01 - 0.84] | 0.49 [ 0.02 - 0.78] |
| Act(Sasaki) vs Fitbit | *-0.20 [-0.63 - 0.23]* | 0.45 [0.34 - 0.56] | 0.57 [0.37 - 0.75] | 0.33 [0.13 - 0.53] | 0.49 [-0.08 - 0.82] | 0.46 [-0.05 - 0.78] |
| ***Moderate*** | | | | | | |
| Act(Uni) vs Act(Sev) ^a^ | 0.83 [0.72 - 0.94] | 0.88 [0.81 - 0.94] | 0.89 [0.67 - 0.93] | 0.77 [0.43 - 0.86] | 0.98 [0.93 - 0.99] | 0.91 [ 0.80 - 0.96] |
| Act(Uni) vs Act(Sasaki) ^a^ | 0.84 [0.72 - 0.96] | 0.76 [0.66 - 0.85] | 0.89 [0.66 - 0.93] | 0.72 [0.35 - 0.82] | 0.96 [0.89 - 0.99] | 0.97 [ 0.91 - 0.99] |
| Act(Sev) vs Act(Sasaki) ^a^ | 0.78 [0.63 - 0.92] | 0.68 [0.56 - 0.80] | 0.87 [0.67 - 0.92] | 0.63 [0.27 - 0.76] | 0.91 [0.74 - 0.97] | 0.80 [ 0.61 - 0.91] |
| Act(Uni) vs Fitbit | *-0.26 [-0.65 - 0.12]* | 0.51 [0.36 - 0.66] | 0.81 [0.56 - 0.89] | 0.66 [0.30 - 0.78] | 0.97 [0.89 - 0.99] | 0.97 [ 0.90 - 0.99] |
| Act(Sev) vs Fitbit | *-0.26 [-0.65 - 0.12]* | 0.55 [0.40 - 0.70] | 0.79 [0.53 - 0.87] | 0.63 [0.26 - 0.76] | 0.92 [0.76 - 0.98] | 0.91 [ 0.76 - 0.97] |
| Act(Sasaki) vs Fitbit | *-0.22 [-0.49 - 0.06]* | 0.51 [0.41 - 0.61] | 0.81 [0.58 - 0.88] | 0.58 [0.27 - 0.70] | 0.98 [0.95 - 1.00] | 0.93 [ 0.86 - 0.97] |
| ***Severe*** | | | | | | |
| Act(Uni) vs Act(Sev) ^a^ | 0.93 [0.83 - 1.02] | 0.46 [0.38 - 0.53] | 0.72 [0.64 - 0.84] | 0.21 [0.09 - 0.45] | 0.96 [0.78 - 0.99] | *-0.18 [-0.58 - 0.30]* |
| Act(Uni) vs Act(Sasaki) ^a^ | 0.47 [0.10 - 0.83] | 0.30 [0.20 - 0.41] | 0.73 [0.40 - 0.85] | 0.27 [0.04 - 0.52] | *-0.29 [-0.83 - 0.52]* | 0.40 [ 0.13 - 0.61] |
| Act(Sev) vs Act(Sasaki) ^a^ | 0.41 [0.01 - 0.80] | 0.42 [0.29 - 0.55] | 0.62 [0.34 - 0.86] | 0.36 [0.05 - 0.66] | *-0.14 [-0.77 - 0.62]* | *-0.12 [-0.66 - 0.50]* |
| Act(Uni) vs Fitbit | *-0.05 [-0.12 - 0.02]* | *0.02 [-0.01 - 0.05]* | 0.55 [0.28 - 0.68] | 0.17 [0.06 - 0.33] | *-0.38 [-0.86 - 0.44]* | *-0.25 [-0.65 - 0.25]* |
| Act(Sev) vs Fitbit | *-0.04 [-0.10 - 0.02]* | 0.11 [0.04 - 0.18] | 0.52 [0.42 - 0.71] | 0.26 [0.07 - 0.42] | *-0.36 [-0.85 - 0.46]* | *-0.31 [-0.78 - 0.37]* |
| Act(Sasaki) vs Fitbit | *-0.13 [-0.27 - 0.01]* | 0.11 [0.02 - 0.19] | 0.49 [0.46 - 0.81] | 0.22 [0.10 - 0.42] | *-0.16 [-0.78 - 0.61]* | *-0.15 [-0.71 - 0.53]* |
| ^a^ Comparison between two criterion measures  Point estimates which did not reach statistical significance, defined here as the 95% confidence intervals excluding 0, are shown in grey italics.  *Act: Actigraph; Uni: Uniform; Sev: Severity ; k: Fleiss’ kappa; CI: confidence interval; r: Pearson correlation coefficient; CCC: Lin’s Concordance correlation coefficient* | | | | | | |
